# Interaction of Male Specific Lethal complex and genomic imbalance on global gene expression in *Drosophila*

**DOI:** 10.1038/s41598-021-99268-y

**Published:** 2021-10-04

**Authors:** Shuai Zhang, Haizhu Qi, Cheng Huang, Lijia Yuan, Ludan Zhang, Ruixue Wang, Yu Tian, Lin Sun

**Affiliations:** 1 Beijing Key Laboratory of Gene Resource and Molecular Development, College of Life Sciences, Beijing Normal University, Beijing, 100875 China; 2 Present Address: Institute of Animal Husbandry and Veterinary Medicine, Beijing Academy of Agriculture and Forestry Sciences, Beijing, 100193 China

**Keywords:** Gene expression, Genomics, Sequencing, Genetics

## Abstract

The inverse dosage effect caused by chromosome number variations shows global consequences in genomic imbalance including sexual dimorphism and an X chromosome-specific response. To investigate the relationship of the MSL complex to genomic imbalance, we over-expressed MSL2 in autosomal and sex chromosomal aneuploids, and analyzed the different transcriptomes. Some candidate genes involved in regulatory mechanisms have also been tested during embryogenesis using TSA-FISH. Here we show that the de novo MSL complex assembled on the X chromosomes in females further reduced the global expression level on the basis of 2/3 down-regulation caused by the inverse dosage effect in trisomy through epigenetic modulations rather than induced dosage compensation. Plus, the sexual dimorphism effect in unbalanced genomes was further examined due to the pre-existing of the MSL complex in males. All these results demonstrate the dynamic functions of the MSL complex on global gene expression in different aneuploid genomes.

## Introduction

Variations in the number of chromosomes can generally be divided into two categories, euploid variation and aneuploid variation^1,2^. The former refers to the increase or decrease of the entire chromosome complement, which produces polyploids or haploids^2^. The latter is usually the addition or loss of individual chromosome or chromosome segments from a diploid to produce aneuploidy^1^. Both euploid variation and aneuploid variation have certain impacts on the survival and development of organisms^3–5^. However, the changes in the number of chromosomes in aneuploidy result in the dosage changes of thousands of genes, leading to imbalances within the genome^6,7^, and having a significant impact on the physiological functions of organisms. Therefore, the influence of aneuploidy on organisms is much greater than that of euploid variation^8,9^. Aneuploidy is usually manifested as ontogenetic abnormalities and premature death, and it is predominantly embryonic lethal in humans^10–12^. Most autosomal aneuploidy in mice have the same early embryonic performance as humans, indicating that aneuploidy seriously affects the early embryonic development stage of animals^7,13^. And aneuploidy is also a common feature of cancer and is present in more than 90% of solid tumors^14–16^.

In various studies of different aneuploid organisms, gene dosage effects referring to the changes in gene expression caused by dosage changes of chromosome fragments have been divided into two different effects. The first one is positive gene dosage effect, in which the expression level of a gene is positively correlated with its dosage^4,17^. The other is the inverse dosage effect, which refers to the inverse relationship between gene expression and the copy number of a trans region (that is, a decrease in gene expression with increased dosage of another chromosome fragment)^18–21^. When the gene copy number increases with the chromosome dosage in aneuploidy, a positive dosage effect will lead to the increase of the expression of this gene, while an inverse dosage effect will result in a decrease. It has been found that the inverse dosage effect is predominant in various aneuploid organisms, including Arabidopsis, maize, Datura, *Drosophila* and human cells^9,18,22–24^. Furthermore, an inverse dosage effect acts across the genome, not only on the varied chromosomes^9,24,25^. This is also the reason why the expression of most genes in aneuploidy tends to decrease with the increase of selected gene dosage^9,19,26,27^.

Dosage compensation refers to the equalized expression of different dosage of X chromosomes in females and males, which is achieved by a twofold up-regulation of the gene expression on the single X chromosome in males to match the gene expression level of the two chromosomes in females^28,29^. It is actually the result of the simultaneous action of the positive dosage effect and the inverse dosage effect^30–33^. The dosage compensation mechanism not only compensates for differences of sex chromosomes, but also occurs for autosomal regions^34^.

The Male-Specific Lethal complex (MSL complex) plays an important role in regulating gene expression during dosage compensation^35–37^, but it is still unclear about the intersection of the effect of changes of chromosomal dosage and the MSL complex on global gene expression in *Drosophila*, as well as the molecular mechanism of how the complex mediates global expression^38^. Compared with the normal females, the gene expression of the single X chromosome in males can be balanced with females through the inverse dosage effect^39^. Studies have found that the inverse dosage effect caused by the single X chromosome in males tends to up-regulate the expression of the whole genome, while the MSL complex recruits histone modifiers, such as MOF, from the autosomes to the X chromosome. The MSL complex prevents over-compensation of the X chromosomes caused by high level histone acetylation, even though the acetylation is generally considered to be related with up-regulation of gene expression^40–42^.

The above studies indicate that the inverse dosage effect plays an important role in the two-fold up-regulation of X chromosome genes, which is modulated by the MSL complex^38^. However, whether the MSL complex promotes or inhibits the inverse dosage effect or their relationship in unbalanced genomes is still unknown. In addition, previous studies have also found that gene expression in female and male *Drosophila* responds differently to genomic imbalance, showing sexual dimorphism, and genes with sex-biased expression are also affected by dosage-sensitive aneuploid effects^34^.

In order to test the interactions between the MSL complex and inverse dosage effect in unbalanced genomes and their related sexual dimorphisms in gene expression, we compared and analyzed the global transcriptomes of different over-expressing MSL2 aneuploids and normal aneuploids, especially the females with de novo assembled MSL complex. The functions of some candidate genes in the early embryogenic stages are further illustrated through high-resolution TSA-FISH technique.

## Results

### Ectopically-expressed MSL complex with autosomal aneuploidy

**Figure 1:**
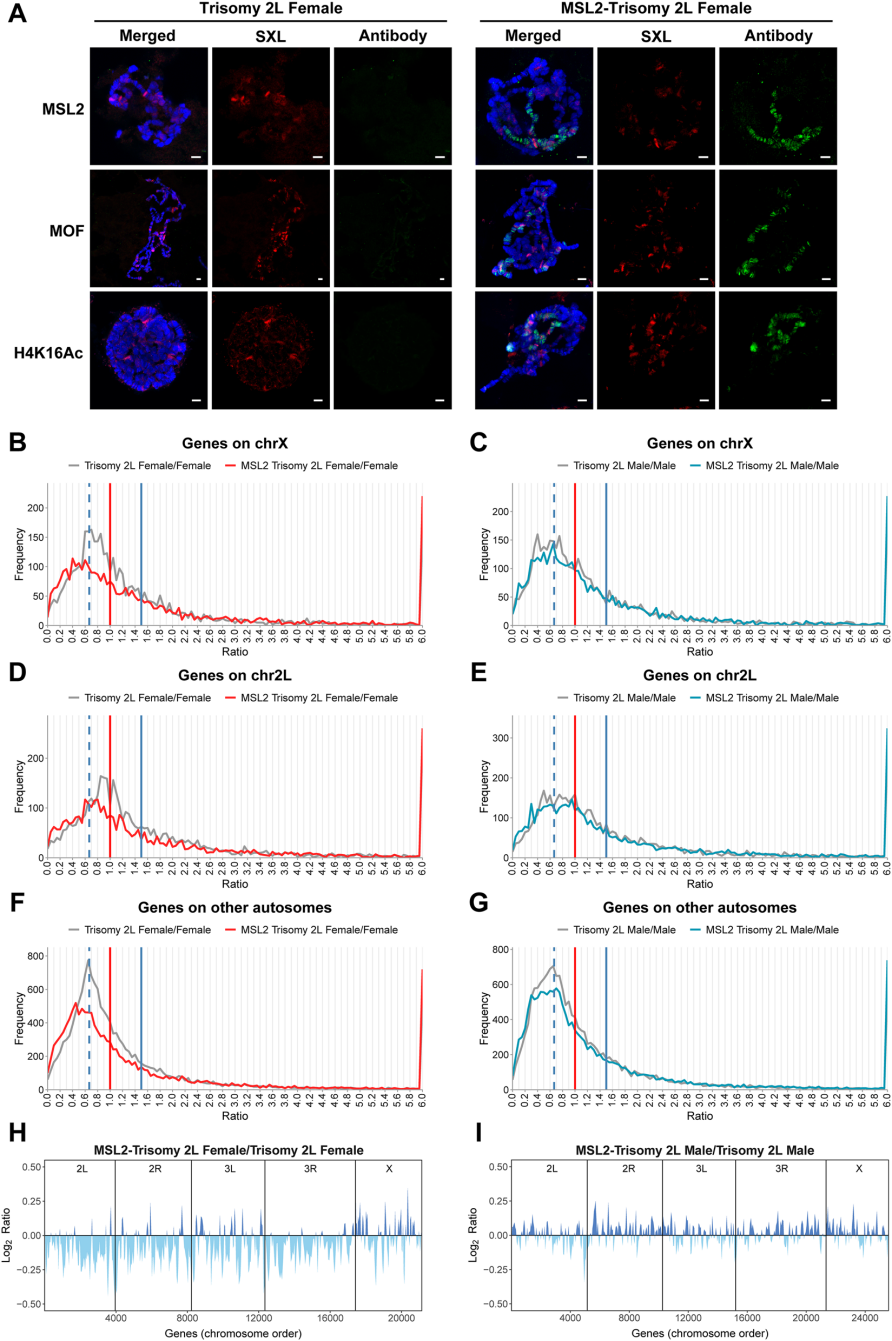
Over-expressed MSL2 in autosomal aneuploidy. (**A**) Immunofluorescence of *Drosophila* polytene chromosomes from third instar larvae of trisomy 2L females (left panel) and MSL2-trisomy 2L females (right panel). The red channel is the signal from SXL and the green channel is the signal from antibodies of MSL complex components. DNA is stained with DAPI in blue. Scale bars, 5 μm. (**B**–**G**) Ratio distributions of gene expression in trisomy 2L and MSL2-trisomy 2L compared with normal diploid in females (**B**, **D**, **F**) and males (**C**, **E**, **G**). All genes are divided into X (**B**, **C**), 2L (**D**, **E**) and other autosomes (**F**, **G**) according to their positions on chromosomes. The vertical red solid line represents the ratio 1.00 (no change), the vertical blue solid line represents the ratio 1.50 [the ratio of gene dosage effects (3/2)], and the vertical blue dashed line shows the ratio 0.67 [the ratio of inverse dosage effects (2/3)]. The global endogenous gene expression patterns of these genotypes were obtained by RNA sequencing. The ratio distributions were generated as described in *Methods* and the frequencies were plotted in bins of 0.05. (**H** and **I**) Distributions of gene expression fold changes along the chromosomes in MSL2-trisomy 2L compared with trisomy 2L in females (**H**) and males (**I**). Each chromosome is separated by a vertical line. Lowess smoothed log2 fold change (log2 ratio) were ordered at equidistance by their chromosomal position. Regions with up-regulated gene expression are shown in dark blue, while regions with down-regulated gene expression are shown in light blue.

In order to identify the interaction between the MSL complex and the effects of genomic imbalance in autosomal aneuploidy, we ectopically expressed the key protein MSL2 in trisomy 2L following the genetic cross shown (Fig. S1, A and B). There is no MSL complex accumulated on the X chromosome in females without MSL2 expression, because of the inhibition by the active Sex Lethal (SXL) protein (Fig. 1A, left panel; Fig. S1E). However, when the MSL2 protein is over-expressed by bypassing the inhibition of SXL, the functional MSL complex is assembled on the X chromosome as confirmed by the immunolocalization of polytene chromosomes from third instar larvae of MSL2-trisomy 2L females using antibody of MSL2, MOF and H4K16Ac (Fig. 1A, right panel). These results indicate that the de novo assembly of the MSL complex will be formed on the X chromosome by bringing the endogenous components from across the genome, like histone acetyltransferase MOF, which is specially associated with high levels of H4K16Ac.

To further determine whether the newly assembled MSL complex induces dosage compensation of X chromosome genes in aneuploid females, we examined the global expression and made ratio distributions of gene expression in trisomy 2L and MSL2-trisomy 2L compared with the normal diploid (Fig. 1B). Randomly selected genes were used to verify the RNA-seq results (Table S1 and S2). This analysis showed that, the gray line of the trisomy 2L female/diploid female is centered around a ratio of 0.67, which is caused by the inverse dosage effect when the 2L chromosome segment is increased to three copies, and the red line with over-expressed MSL complex does not show any movement to the right side, on the contrary, it produces more down-regulations to the left side with a peak around 0.45 (Fig. 1B; Kolmogorov–Smirnov (K-S) test *P* = 3.27e−9). The results showed that the majority of X chromosome gene expression levels do not exhibit an increased trend when the MSL complex is formed on the X chromosome in trisomy 2L aneuploidy females, which means that the MSL complex does not induce dosage compensation of X chromosome genes in aneuploidy females even with increased H4K16Ac levels (Fig. 1A), coincident with the previous results in normal diploids^40^.

In aneuploids, when the inverse dosage effect and gene dosage effect simultaneously influence the gene expression on the varied chromosome segments, like the genes on chromosome 2L in trisomy 2L, dosage compensation occurs, meaning no expression changes even though the copy number of 2L has been increased to three (Fig. 1D, gray line). Interestingly, the de novo formed MSL complex on the X chromosome in MSL2-trisomy 2L females bring the expression levels of 2L genes to a lower ratio centered around 0.67 when compared with the normal diploid (Fig. 1D, red line; K-S test *P* = 8.34e−14). It is concluded that when the MSL complex is assembled on the X chromosome, it actually takes some modifier factors including MOF from the autosomes to the X chromosome, contributing to a down-regulated expression pattern of 2L genes.

This dynamic function of the MSL complex in the aneuploidy genome was also confirmed by the changes of other autosomal genes, producing a lower ratio distribution around a ratio of 0.4 as well (Fig. 1F, red line; K-S test *P* = 0), even though other autosomal genes are already down-regulated to 2/3 caused by the inverse dosage effect in trisomy 2L aneuploidy.

Meanwhile, the distributions of trisomy 2L with and without MSL2 overexpression compared with normal diploid in males showed distinct patterns on genome-wide genes including X chromosome genes, 2L genes and other autosomal genes (Fig. 1C,E,G; K-S test *P* = 0.0205, 3.00e−3 and 1.59e−8, respectively) compared with respective ratio distributions in females (Fig. 1B,D,F). When the supernumerary MSL complex is assembled in aneuploid males, it is shown that almost all of the genes in the genome do not display any significant changes between MSL2-trisomy 2L and trisomy 2L compared with normal diploid males, which is probably because of the pre-existing presence of the endogenous MSL complex on the X chromosome.

The distribution of global gene expression fold changes along chromosomal regions was also analyzed when comparing MSL2-trisomy 2L with trisomy 2L in females and males (Fig. 1H,I). It is also found that the regions that are originally reduced by an inverse dosage effect (autosomes except for 2L and chromosome X) are further decreased significantly in MSL2-trisomy 2L females (Fig. 1H), demonstrating that there is an interaction between the action of the MSL complex and a genomic imbalance effect. The X chromosome of trisomy 2L females with ectopically assembled MSL complex is not twofold up-regulated, although its distribution is significantly different from that of other autosomes (Fig. 1H), indicating that the assembly of the MSL complex cannot directly mediate the up-regulation of dosage compensation. In males, however, the magnitudes of alternating up-regulated and down-regulated regions are both small (Fig. 1I). All these results suggest interactions of the effect of aneuploidy and the MSL complex will influence global gene expression. Furthermore, the specific response of the X chromosomal genes especially in females and the sexual dimorphism caused by the MSL complex in aneuploids could be identified (Fig. 1H,I).

### Ectopically-expressed MSL complex with sex chromosomal aneuploidy

**Figure 2:**
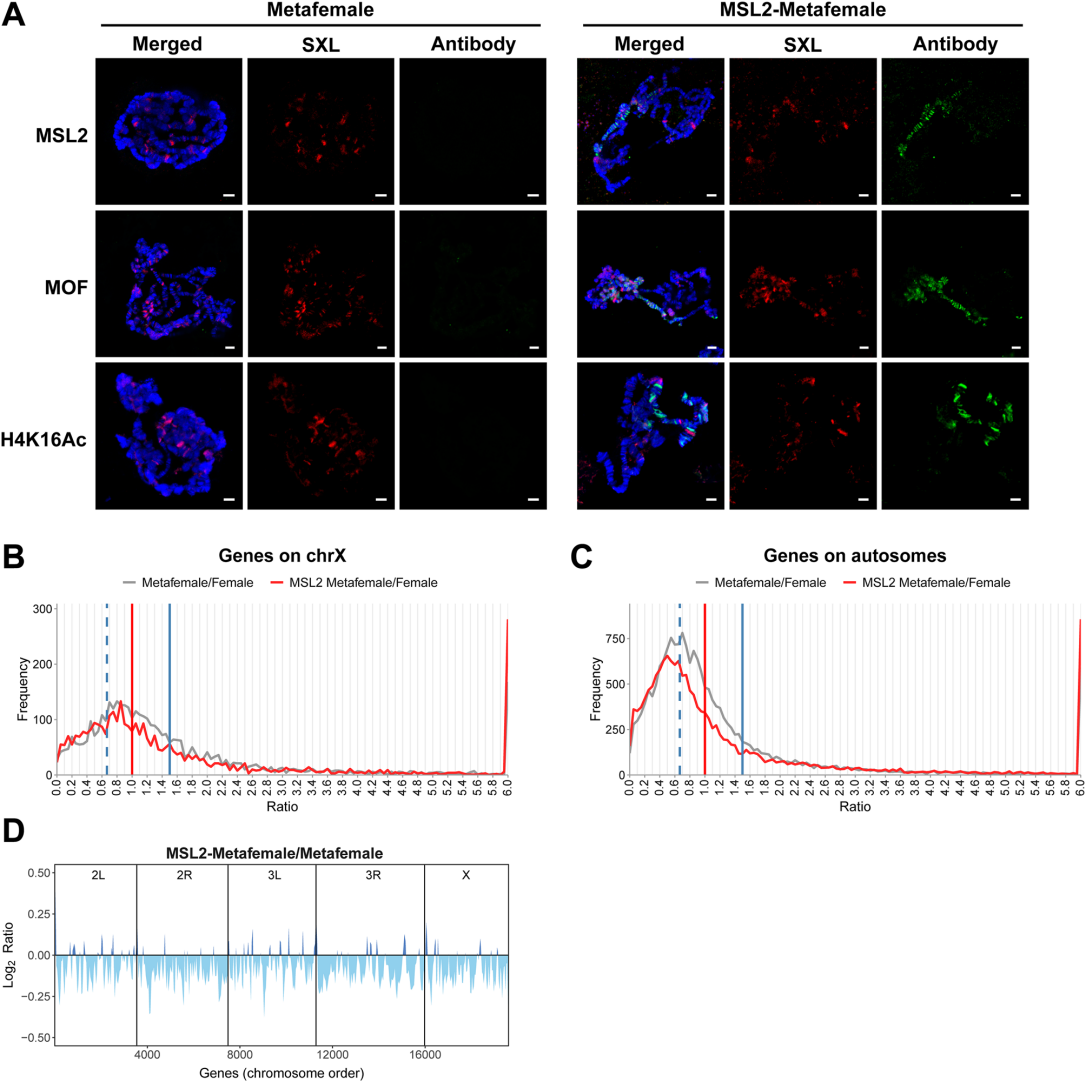
Over-expressed MSL2 in sex chromosomal aneuploidy. (**A**) Immunofluorescence of *Drosophila* polytene chromosomes from third instar larvae of metafemales (left panel) and MSL2-metafemales (right panel). The red channel is the signal from SXL and the green channel is the signal from antibodies of MSL complex components. DNA is stained with DAPI in blue. Scale bars, 5 μm. (**B**, **C**) Ratio distributions of gene expression in metafemale (XXX) and MSL2-metafemale compared with normal diploid. All genes are divided into X (**B**) and autosomes (**C**) according to their positions on chromosomes. The vertical red solid line represents the ratio 1.00 (no change), the vertical blue solid line represents the ratio 1.50 [the ratio of gene dosage effects (3/2)], and the vertical blue dashed line shows the ratio 0.67 [the ratio of inverse dosage effects (2/3)]. The frequencies were plotted in bins of 0.05. (**D**) Gene expression changes along *Drosophila* chromosomes between MSL2-metafemale and metafemale. Each chromosome is separated by a vertical line. Lowess smoothed log2 fold change (log2 ratio) were ordered at equidistance by their chromosomal position. Regions with up-regulated gene expression are shown in dark blue, while regions with down-regulated gene expression are shown in light blue.

We also investigated the effects of the MSL complex and genomic imbalance in sex chromosomal aneuploidy (Fig. 2). When the X chromosome is increased to three copies in metafemales (XXX; AA), the majority of genes on the X chromosomes still display dosage compensation when compared with the normal diploid females (Fig. 2B, gray line). The inverse dosage effect produced by the extra copy of the X chromosome influenced the autosomal expression levels to a ratio distribution centered around 0.67 (Fig. 2C, gray line). To test and verify whether the dynamic functions of MSL complex found in autosomal aneuploidy are still valid in sex chromosomal aneuploidy, the complex is also assembled on the X chromosomes in metafemales with over-expressed MSL2 protein, which is confirmed by the immunostaining with several antibodies (Fig. 2A).

Based on the ratio distribution of X chromosome genes (Fig. 2B), it is found that the de novo MSL complex formed in metafemales does not give rise to any obvious upregulation of X chromosome genes to ratios over 1.0, which means no dosage compensation is induced by its presence on the X chromosome. Moreover, some genes have been down-regulated compared with the gray line even with an accumulated level of H4K16Ac on the X chromosomes (Fig. 2A), coincident with the above data of MSL2-trisomy 2L females. Comparing the expression of X chromosome genes in MSL2-metafemales with the 2L genes in MSL2-trisomy 2L females (Fig. 1D, red line), it is found that the ratio distribution of X chromosome genes in MSL2-metafemales (Fig. 2B, red line; K-S test *P* = 9.03e−6) showed less changes, indicating the different responses of the MSL complex to genomic imbalance between sex chromosome and autosome.

With the formation of the MSL complex on the X chromosomes in metafemales, the other endogenous components are also brought from the autosomes to the X chromosomes. In this situation, the autosomal genes are also found to show decreased expression levels even though the inverse dosage effect has already produced a 2/3 down-regulation as the above results concluded from autosomal aneuploidy (Fig. 2C; K-S test *P* = 0), which is coincident with the distribution of global gene expression fold changes along chromosomal regions when comparing MSL2-metafemale with metafemale (Fig. 2D). Based on the above results, the dynamic functions of MSL complex are further confirmed by the analyses of global gene expression in X chromosome aneuploidy.

### Sexual dimorphism in MSL2-aneuploidy

It has been found that when an extra 2L chromosome arm is added into the genome in *Drosophila*, there are significant differences between the sexes^34^. In addition, the expression levels of sex biased genes with significant differences between normal females and males, and genes with no significant differences as non-sex biased genes were further analyzed to identify the sexual dimorphism in comparisons between trisomy aneuploid with normal diploid. All these results demonstrated a correlation between the inverse dosage effect and different sexes^34^.

**Figure 3:**
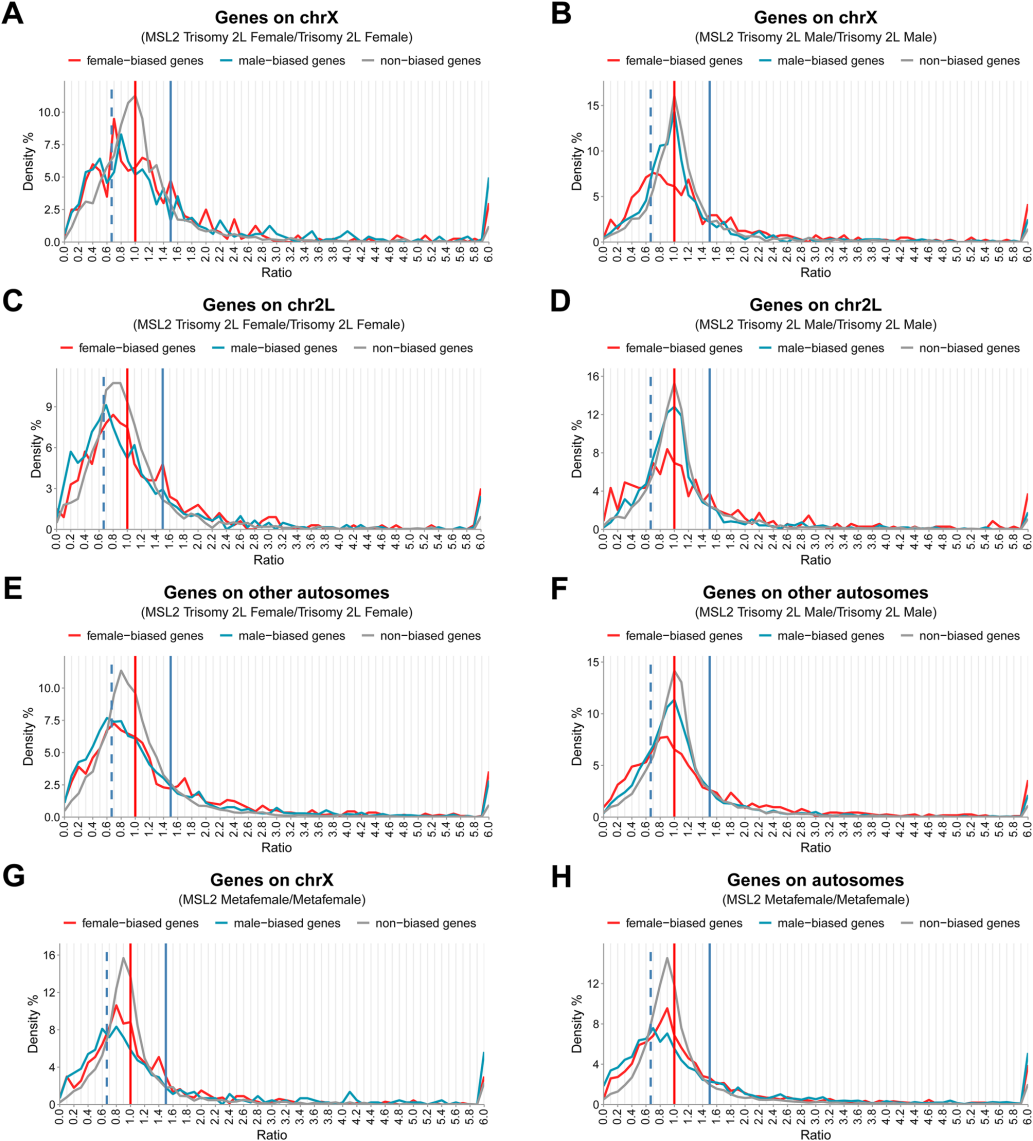
Ratio distributions of sex-biased genes and non-sex-biased genes. (**A**–**F**) Ratio distributions of sex-biased and non-sex-biased transcript isoforms in MSL2-trisomy 2L compared with trisomy 2L in females (**A**, **C**, **E**) and males (**B**, **D**, **F**). All genes are divided into X (**A**, **B**), 2L (**C**, **D**) and other autosomes (**E**, **F**) according to their positions on chromosomes. (**G**, **H**) Ratio distributions of sex-biased and non-sex-biased transcript isoforms in MSL2-metafemale compared with metafemale. All genes are divided into X (**G**) and autosomes (**H**) according to their positions on chromosomes. The vertical red solid line represents the ratio 1.00, the vertical blue solid line represents the ratio 1.50, and the vertical blue dashed line shows the ratio 0.67. The percentages of frequencies were plotted in bins of 0.1.

In order to further determine the role of the MSL complex in sexual dimorphism based on its special role between females and males in these unbalanced genomes, ratio distributions of genes with sex-biased and non-sex-biased expressions in MSL2-trisomy 2L compared with trisomy 2L are also performed (Fig. 3A–F).

It is found that sex-biased genes on the X chromosome showed a decreased change with the center ratio around 0.67 (Fig. 3A, red line and blue line) in autosomal aneuploidy when de novo MSL complex is assembled on the X chromosome in females. More interestingly, besides the highest peaks around 0.67, some other peaks around 1.5 are also detected in respective comparisons. In contrast, non-biased genes on the X chromosome do not exhibit any obvious change with a highest peak still focused on a ratio of 1.0 (Fig. 3A, gray line). Similar results are also found on the ratio distributions of male-biased and non-biased genes in the comparison of males (Fig. 3B, blue line and gray line). However, the female-biased genes on the X showed a different pattern, in which there are two different obvious peaks on the both sides of ratio 1.0, the highest peak around 0.67 representing the majority of genes that are decreased and a smaller peak around 1.25 meaning some other genes are increased (Fig. 3B, red line). When comparing the genes on the X chromosome between the sexes, it was found that both female-biased genes and non-biased genes have a similar response to the newly formed MSL complex in trisomy 2L aneuploids (Fig. 3A,B, red line and gray line), in which female-biased genes are decreased and non-biased genes are not changed. But the male-biased genes on the X expressed different consequences in different sexual comparisons when the complex is combined with the inverse dosage effect in aneuploids (Fig. 3A,B, blue line). All these results illustrated that sex-biased genes on the X exhibit sexual dimorphism in these unbalanced genomes when the MSL complex is built up on the X chromosomes.

The sex-biased genes on the autosomes including 2L chromosomes and other autosomes are also compared in each sex. It is concluded that all the genes are brought to a lower expression level with the presence of the MSL complex in females (Fig. 3C,E), and the female-biased genes in males as well (Fig. 3D,F, red line). And it is also found that female-biased autosomal genes express more decreased levels in females than in male. Furthermore, male-biased genes in females are more sensitive to the newly formed MSL complex and exhibit lower ratio distributions than female-biased genes (Fig. 3C,E). However, the male-biased and non-biased genes do not show any response to expressed MSL complex in males (Fig. 3D,F, blue and gray lines).

When sex-biased genes on the X chromosome are compared with sex-biased autosomal genes, the distinct distribution pattern of X chromosome genes could be found in both sexes, especially in the females (Fig. 3,A,C,E). It is concluded that the X chromosome genes show a distinctive response to the MSL complex in trisomy 2L genomes.

Similar analyses are also performed in the comparisons of MSL2-metafemales with metafemales to further determine the functions of MSL complex in aneuploids (Fig. 3G,H). It is demonstrated that male-biased genes showed stronger reaction to the new MSL complex in this genome than female-biased and non-biased genes (Fig. 3G,H), which is a similar pattern as the comparisons of autosomal genes in MSL2-trisomy 2L (Fig. 3C,E). Also, sex-biased genes on the X chromosome display more response to the new complex than the genes on the autosomes (Fig. 3G,H). The different consequences of gene expression changes between the X chromosome and the autosomes caused by the MSL complex are also identified in this unbalanced genome with one extra copy of the X chromosome.

### Gene coexpression networks in MSL2-aneuploidy

**Figure 4:**
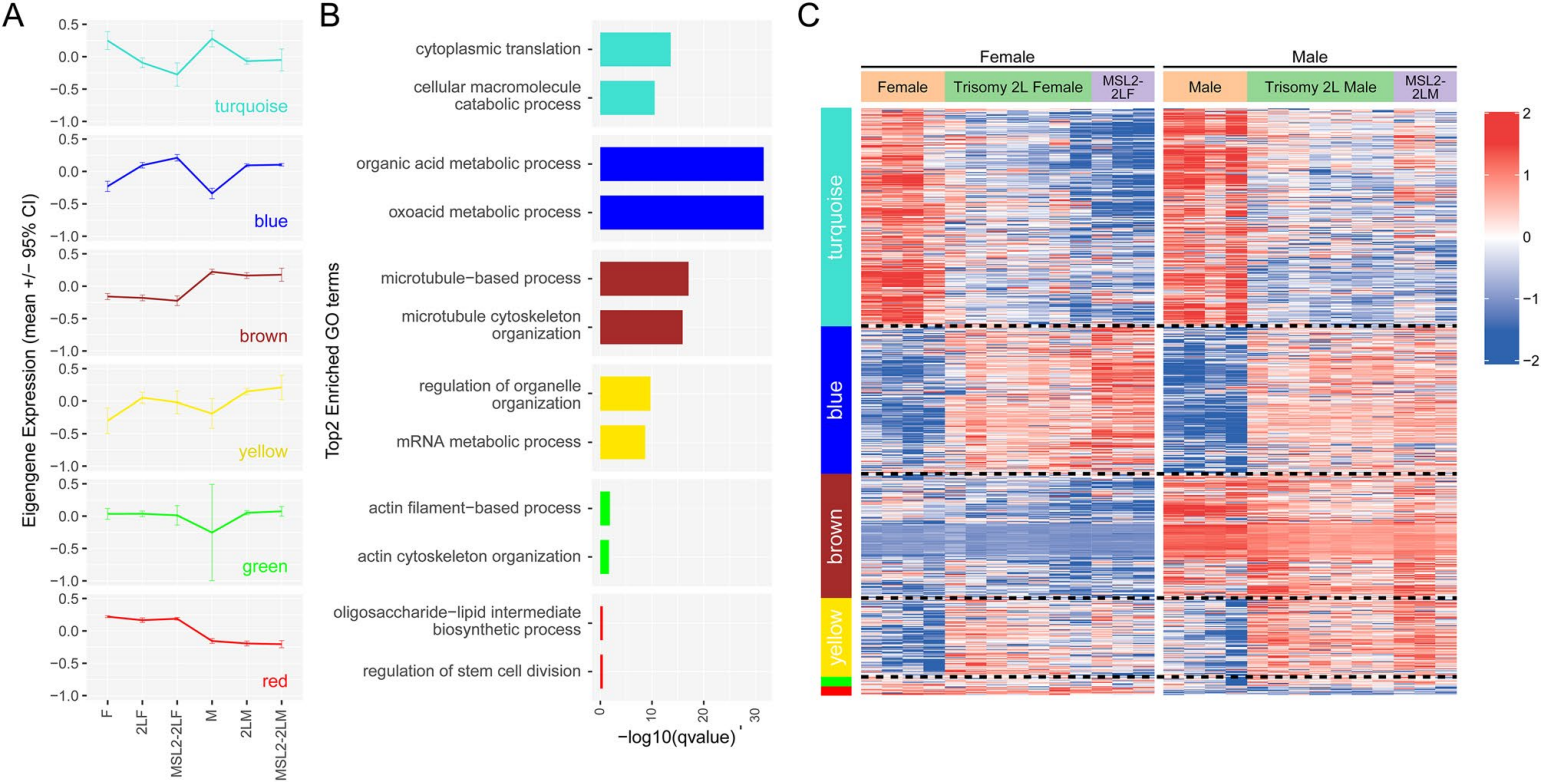
Weighted gene coexpression network analysis (WGCNA) of autosomal aneuploidy *Drosophila*. (**A**) Dot and line plots showing the eigengene expression (mean ± 95% confidence interval) of each gene-coexpression module by genotypes in autosomal aneuploidy groups. The modules are ordered according to the number of genes they contain. F, female; 2LF, trisomy 2L female; MSL2-2LF, MSL2-trisomy 2L female; M, male; 2LM, trisomy 2L male; MSL2-2LM, MSL2-trisomy 2L male. (**B**) Top two GO term enrichments for each module. (**C**) Heatmap showing the expression level of genes in every coexpression module in autosomal aneuploidy groups. Each column is a biological repetition and each row is a gene. Hierarchical clustering is carried out only within the module and genotype groups. The horizontal dotted lines divide the four largest modules. The 10,000 genes with the highest average expression were involved in the coexpression network analysis. Gray modules are not considered in the plots because they represent unclustered genes.

To gain a comprehensive understanding of gene expression relationships in unbalanced genomes and the functions of MSL complex, we performed weighted gene coexpression network analysis (WGCNA). We identified 6 independent gene-coexpression modules (Turquoise, Blue, Brown, Yellow, Green and Red) in the trisomy 2L dataset, and the number of genes they contained decreases progressively from 3,000 to 100 (Fig. 4A). The eigengene expression of the biggest Turquoise module is significantly decreased in trisomy 2L female and trisomy 2L male, and further decreased in MSL2-trisomy 2L females, but the overexpression of MSL2 has no effect on trisomy 2L males (Fig. 4A). The Blue module is close to the mirror image of the Turquoise module, in which the eigengene expression is increased in trisomy 2L, and further increased in MSL2-trisomy 2L female. These two modules show that most genes are affected by the inverse dosage effect in aneuploidy and the MSL complex seems to play a similar role to the effect of genomic imbalance, which is weakened in males. The function of the Turquoise module is mainly enriched in cytoplasmic translation and cellular macromolecule catabolic process (Fig. 4B). We speculate that the interaction between the MSL complex and genomic imbalance involves various transcription factors, which are considered to be dosage-sensitive, to regulate gene expression, and we verified that this module contained a relatively high number of transcription factors (Fisher’s exact test pvalue = 0.042). Another module containing a high number of transcription factors is the Yellow module (Fisher’s exact test pvalue = 5.98e−7). It displays a different pattern in that the overexpressed MSL2 produced different responses in female and male based on the dosage effect, specifically down-regulation in aneuploid female and up-regulation in aneuploid male (Fig. 4A). This module is enriched for mRNA metabolic process and regulation of organelle organization. The Brown and Red module represent two major categories of genes that are expressed with sex bias. The Green module is a subset of genes that showed a large variation in males. The expression pattern of eigengenes can be perfectly reproduced at the level of individual genes and samples (Fig. 4C).

We also performed WGCNA in our metafemale dataset and found 5 modules (Fig. S2). The largest module shows a decrease trend in metafemales and a further decline in MSL2-metafemales (Fig. S2A), similar to the autosomal aneuploidy. Its functions are enriched in ubiquitin-dependent protein catabolic process and modification-dependent macromolecule catabolic process (Fig. S2B). It can be noted that the modules showing an inverse dosage effect (Turquoise) in autosomal aneuploidy and sex chromosomal aneuploidy both involve macromolecular complexes, which indicates the effects of genomic imbalance maybe related to the altered stoichiometry of the components of macromolecule complexes. Similarly, this module is enriched with more transcription factors (Fisher’s exact test *P* value = 5.83e−21). In general, the gene coexpression networks in autosomal aneuploidy and sex chromosomal aneuploidy displays a certain similarity about the interaction of MSL complex and genomic imbalance, and the regulatory of transcription factors. The effect of genomic imbalance may result by the altered relative expression of members of complexes although modulation of transcription factors will have a cascading effect.

### Differentially expressed genes in MSL2-aneuploidy

**Figure 5:**
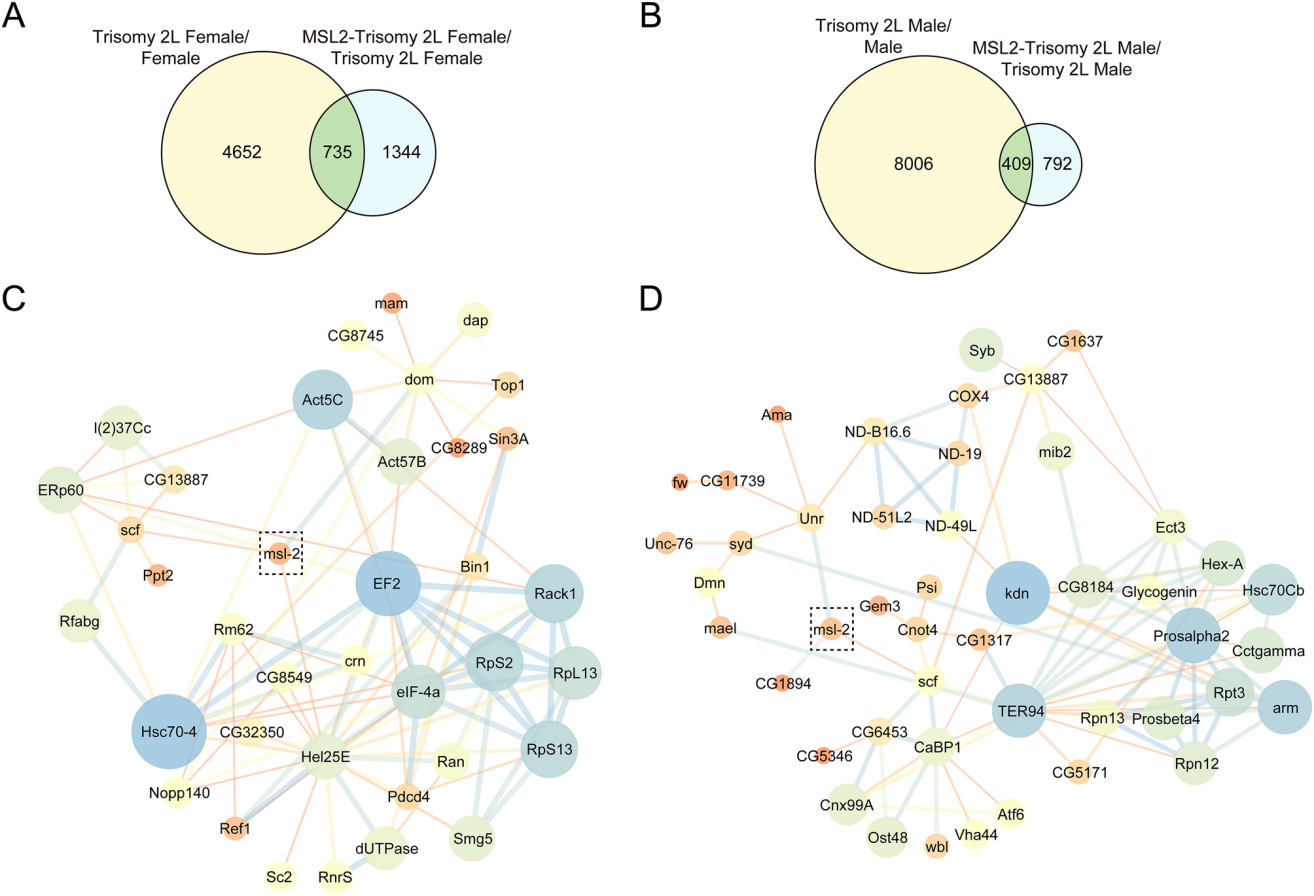
Protein–protein interaction (PPI) networks of the genes affected by both aneuploidy and MSL complex. (**A** and **B**) Venn diagrams show the number of differentially expressed genes in MSL2-trisomy 2L/trisomy 2L females (**A**) and males (**B**). Genes with padj lower than 0.05 are selected as differentially expressed genes. (**C** and **D**) The differentially expressed genes from trisomy 2L/normal diploid and MSL2-trisomy 2L/trisomy 2L in females (**C**) and males (**D**) are intersected to generate the PPI networks. Only common genes which are associated with *msl-2* and have the shortest path less than two edges in female or three edges in male are shown. The nodes in dotted box show the gene *msl-2*. The thickness of the edge refers to combined score, and the size of the node is representative of degree.

To determine the variation of aneuploidy and the effect of the MSL complex on gene expression in aneuploids, we analyzed the differential gene expression in RNA sequencing data. The number of differentially expressed genes (padj < 0.05) in comparisons of trisomy/normal diploids and MSL2-trisomy/trisomy are shown in Venn diagrams (Fig. 5A,B; Fig. S3, A and B). It is illustrated that the inverse dosage effect produced by the extra copy of 2L in trisomic aneuploids occurs for much more differentially genes in males (male = 8415) than in females (female = 5387) (Fig. 5A,B), which is coincident with previous results of sexual dimorphism in unbalanced genomes. It is indicated that males are less able to maintain genomic expression homeostasis when disturbed by autosomal aneuploidy. A substantial number of genes are differentially expressed in both trisomy 2L males and females (3337, *P* value < 2.2 e−16), indicating the similarity of the effects of the same chromosome trisomy on organisms.

When de novo MSL complex is assembled in trisomy 2L females, there is a total of 2079 differentially expressed genes induced by the presence of the MSL complex and inverse dosage effect, 735 of which are simultaneously regulated by the inverse dosage effect only (Fig. 5A). Interestingly, the number of total differentially expressed genes influenced by these two factors together in males is 1201, which is also about half the number as that in females (Fig. 5B). It is coincident with the previous results of RNA-seq that the de novo MSL complex affects much more global expression levels in females than in males. Furthermore, the number of common regulated genes in males is 409 by the inverse dosage effect, which is almost 1/3 of the total differentially expressed genes (409/1201) in males (Fig. 5B). More interestingly, a similar ratio (735/2097) is also identified in females when the complex is formed (Fig. 5A). Based on these results, the relationship between the MSL complex and the global gene expressions in aneuploids is further confirmed.

We also compared the gene expression of sex-chromosomal trisomy (metafemale) with that of autosomal trisomy, and found that gene expression of metafemales was less affected than that of autosomal aneuploidy (Fig. S3A). This phenomenon may be due to the fact that autosomal aneuploidy is more detrimental to organisms. The effect of overexpression of MSL2 appears to be greater in both metafemales and trisomy 2L females than in males (Fig. S3B), coincident with the previous results that the de novo MSL complex showed greater effect in females than in males.

### Global expression patterns of RNA transcripts in MSL2-aneuploidy embryos

The inverse dosage effect produced by genomic imbalance in aneuploids exhibits global regulation on gene expression, and the ectopically expressed MSL2 also showed extensive impact in aneuploidy. In order to determine the relationship of the MSL complex and the inverse dosage effect, protein interaction analysis was further performed on the common differentially expressed genes in trisomy 2L and MSL2-trisomy 2L. Since there may be natural differences between sexes, females and males are analyzed separately (Fig. 5C,D). A protein–protein interaction (PPI) network diagram was drawn containing proteins within 2–3 edges of *msl-2* at the shortest distance, with *msl-2* as the center. In the protein interaction network, genes with high expression level, direct interaction with MSL-2, and indirect interaction with high degree of connectivity were selected as candidate genes. In addition, a glucose metabolism-related gene *Mal-A3* and a non-coding RNA *CR45570* were selected, and the components of MSL complex were also included in the subsequent analysis. All detailed information about these genes and their functions are listed (Table 1 and S3).

**Table 1:** The expression of candidate genes in embryo-FISH. Up- or down-regulation in the table represent significant differences (Student’s *t* test *P* < 0.05) in gene expression based on the analysis of relative fluorescence intensity of FISH.

Genes	Chromosome	Function	Stage 1–5		Stage 6–11		Stage 12–13		Stage 14–17	
			Trisomy 2L/normal	MSL2-Trisomy 2L/Trisomy 2L	Trisomy 2L/normal	MSL2-Trisomy 2L/Trisomy 2L	Trisomy 2L/normal	MSL2-Trisomy 2L/Trisomy 2L	Trisomy 2L/normal	MSL2-Trisomy 2L/Trisomy 2L
*Hel25E*	2L	ATP-dependent RNA helicase WM6; Required for mRNA export out of the nucleus	–	–	Up	–	–	Down	Up	–
*RpS2*	2L	40S ribosomal protein S24; Structural constituent of ribosome; Nucleotide binding	Down	Down	Down	Down	–	Down	–	Down
*msl-1*	2L	Components of MSL complex; DNA binding; organization of MSL complex	Down	–	Down	Down	Down	Down	–	Down
*mle*	2R	Components of MSL complex; 3'–5' RNA/DNA helicase activity	–	–	Down	–	–	–	–	–
*Mal-A3*	2R	Maltase A3; cation binding; catalytic activity; Alpha-1,4-glucosidase activity; Belongs to the glycosyl hydrolase 13 family	Down	–	Down	–	Down	Down	–	Down
*TER94*	2R	Transitional endoplasmic reticulum ATPase; Golgi stacks fragmentation and reassembly; Transitional endoplasmic reticulum formation	Down	–	UP	Down	Down	–	Down	Down
*ERp60*	2R	Protein disulfide isomerase activity; Protein folding; Cell redox homeostasis	Down	Up	Down	–	Down	Down	–	Down
*Vha44*	2R	V-type proton ATPase subunit C; Acidify Intracellular compartments in eukaryotic cells	Down	Up	Down	Up	Down	–	Down	–
*scf*	3L	Supercoiling factor (*scf*) encodes two polypeptides: DCB45 and its N-teminally truncated form Scf	Down	–	Down	–	Down	–	Down	–
*Hsc70–4*	3R	Heat shock 70 kDa protein cognate 4; Chaperone binding; unfolded protein binding; ATPase activity	–	–	Down	Up	Down	Up	Down	Up
*CG1894*	3R	Histone acetyltransferase activity	Down	Up	Down	Down	Down	–	–	Down
*CR45570*	3R	Unknown	Down	Down	Down	Down	–	Down	–	Down
*roX1*	X	Components of MSL complex; Long non-coding RNA on the X 1	Down	–	Down	Up	Down	Up	Down	Down
*mof*	X	Components of MSL complex; H4-K16 specific histone acetyltransferase activity	Down	–	–	–	–	Down	–	Down
*ND-B16.6*	X	NADH dehydrogenase (ubiquinone) B16.6 subunit, isoform A; NADH dehydrogenase activity	Down	Down	–	Down	Down	Down	–	Down
*Ran*	X	GTP-binding nuclear protein Ran; GTPase involved in nucleocytoplasmic transport; proteins and RNAs import and export from the nucleus	Down	–	Down	Down	–	Down	Up	Down

**Figure 6:**
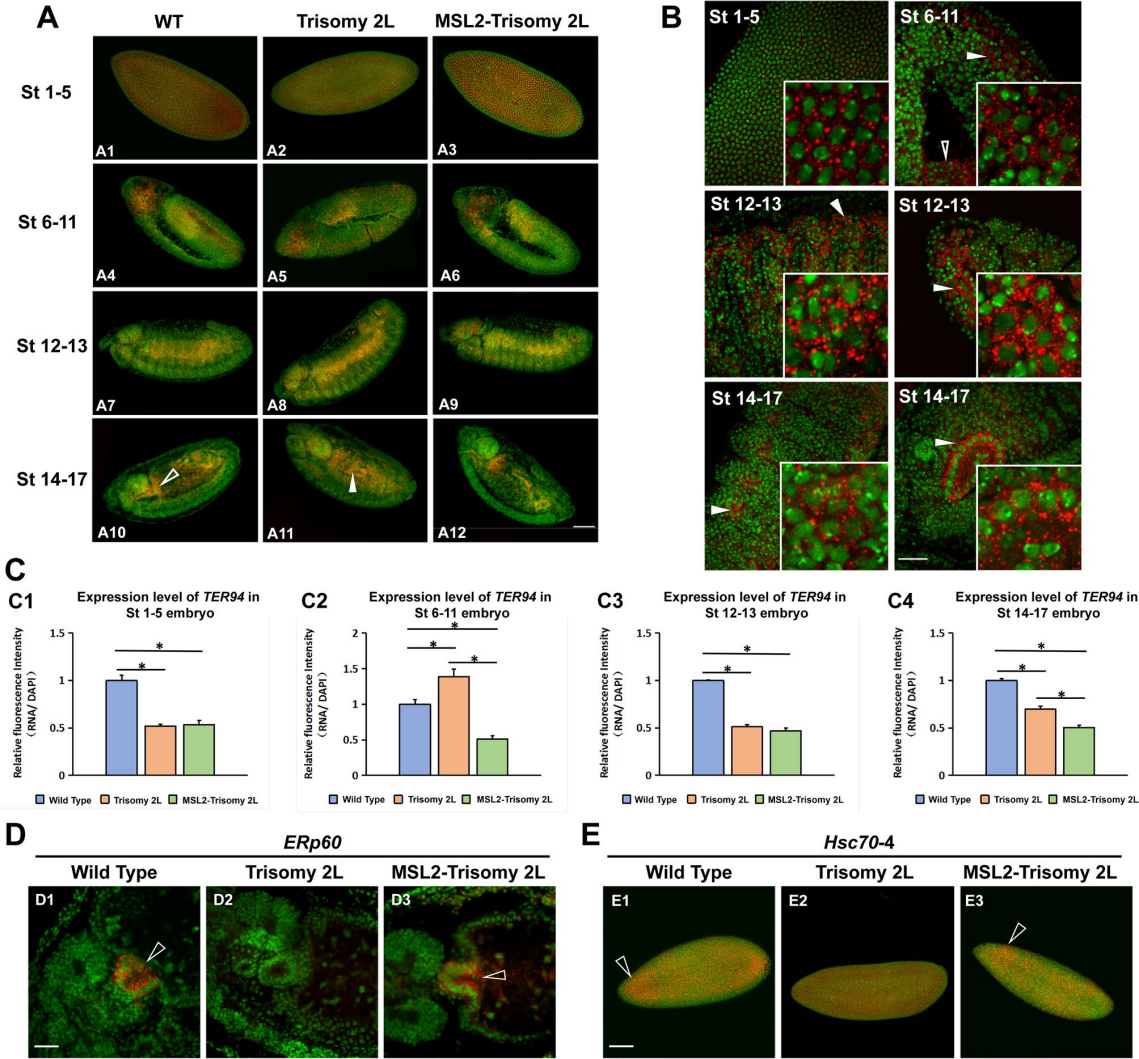
Embryo-FISH. (**A**–**C**) Embryo-FISH of candidate gene *TER94*. (**A**) *TER94* RNA expression patterns in entire embryo. The genotype of the sample is shown in the horizontal axis above, and the development stage of the sample is shown in the left vertical axis. The red pseudo-color is the signal from probe and the green pseudo-color is the signal from nucleus. Scale bars, 80 μm. (**B**) *TER94* RNA subcellular location patterns in wild type embryos. The image shown at the bottom right shows a locally enlarged subcellular localization pattern. Scale bars, 30 μm. (**C**) Relative fluorescence intensity of *TER94* RNA signal in trisomy 2L and MSL2-trisomy 2L compared with normal diploid. The relative fluorescence intensity is determined by comparison with DAPI signal. The expression of wild-type embryos at each stage of development are set at 1. Asterisk denotes a p-value < 0.05 by two-tailed Student’s t tests. (**D**–**E**) Changes of transcript localization of *ERp60* (**D**) and *Hsc70-4* (**E**) in trisomy 2L and MSL2-trisomy 2L. Scale bars, (**D**) 30 μm; (**E**) 80 μm.

Because the aneuploid embryos are associated with some detrimental developmental problems, TSA (Tyramide Signal Amplification)-FISH technique was used to analyze the expression patterns of MSL complex components and candidate genes in *Drosophila* embryos, so as to reveal the mechanism of action of MSL complex and its target factors in aneuploidy (Fig. 6). Furthermore, the quantitative analyses of these RNA transcripts are further tested to determine their functions and validate the global changes of RNA-seq (Fig. S4).

As for MSL complex components, the expression range of roX1 is the widest and transcript localization is found almost throughout the embryo. The subcellular location pattern of roX1 is accumulated in a subset of nuclei, and MSL-1 and MOF show perinuclear localization, while MLE has a cytoplasmic localization (Fig. S4). The expression level of MLE is barely affected in aneuploidy and the ectopic expression of MSL2, while other components of MSL complex are changed significantly. The decrease of the expression level of MOF, a histone acetyltransferase, in early embryos may affect the expression of other genes in the same developmental period. The expression of *roX1* and *msl-1* are inhibited in aneuploidy at most stages of trisomic embryo development. As an important platform for the targeting and assembly of the MSL complex, the decreased expression levels of these two components may lead to the decrease of the overall dose of the MSL complex in the embryo. After ectopic expression of the MSL2 protein, the expression levels of MSL components were affected to varying degrees and most of them were inhibited by the overexpression of MSL2, except *mle* that does not respond to the dosage change of MSL2 (Table 1).

For the candidate target genes, the most prevalent localization pattern is perinuclear localization and cytoplasmic localization. 2L aneuploidy resulted in a significant reduction in expression levels of 75% of detected genes, and 67% of these genes returned to normal expression levels in later embryonic development (Table 1). The ectopic expression of MSL2 can further lower the expression of most detected genes in the embryonic stage, especially in the later stage of development, on the basis of trisomy. In addition, the transcripts of all detected genes were significantly distributed in the brain, midgut and salivary glands, suggesting that MSL2 can interfere with the physiological function and tissue structure of an aneuploid nervous system and digestive system by regulating the expression of MSL complex components and candidate factors (Fig. S4). More interestingly, aneuploidy and the MSL complex affect the transcript localization of *ERp60* and *Hsc70-4* (Fig. 6D,E).

Based on the results of TSA-FISH, ectopically expressed MSL2 may regulate the expression of the candidate genes involved in the interaction of genomic imbalance and the MSL complex, expand the regulatory range of gene expression in aneuploidy, and mediate a variety of biological processes, such as organic metabolism and cell apoptosis, so as to affect the abnormal phenotypes of aneuploidy. The results of TSA-FISH illustrate that the effects described above for larvae also generally occurs in the embryonic stages and that the effects can be visualized on the cellular level with absolute as well as relative quantification.

## Discussion

It has been found that altering the dosage of individual chromosomes has more detrimental effect on the phenotypes than changing the whole set of chromosomes due to genomic imbalance^5^, a complicated phenomenon involving a stoichiometric relationship of regulatory molecules on the varied chromosomal segments and the remainder of the genome^5,43^. And the relative dosage of chromosome segments is vital for the global gene expression, in which any target gene could be modulated by different regions of the genome. The gene dosage effects are produced by variations in the number of chromosomes, any chromosomal segments, or even some individual genes in genomic imbalance, including gene dosage effects and inverse dosage effects, which is the predominant response in aneuploidy^19,22,44^. It has been concluded that the global inverse dosage effect caused by trisomy 2L^34^ or metafemales^21^ exhibited similar decreased expression patterns across the unbalanced genomes, and only the genes on the varied chromosomes show dosage compensation, which is contributed by the interactions between the gene dosage effect (3/2) and the inverse dosage effect (2/3) simultaneously.

In normal males, the X chromosome is a modified monosomic situation that has evolved to be viable, and the inverse dosage effect caused by the half-dose X chromosome plays a role to modulate gene expression to achieve compensation. In order to balance the expression pattern across the genome, the MSL complex, only present and accumulated on the X chromosome in normal male *Drosophila* (Fig. S1E) concentrates histone modifications such as H4K16Ac, and H3S10 that typically are associated with increased gene expression^29,45–48^. It also recruits molecular inhibitors to counteract these up-regulated genes to maintain the doubled regulation in males^21,30,40^. To date, it is concluded that the MSL complex and the inverse dosage effect produced by the X chromosome monosomy collaborate together to achieve the dosage compensation of X chromosome and the balance of the whole genome^21,26,30,40,49^.

Interestingly, some results have shown that ectopically assembly of the MSL complex on the female X chromosomes in normal diploid does not result in twofold up-regulation of gene expression even with an increased level of H4K16Ac modification, indicating the repressive mechanism of the complex to counteract high level of histone modifications^50–52^. And this repressive function is also confirmed by the different consequences of over-expressed MOF in females and males in which MOF increases expression only the absence of the MSL complex^40^. Meanwhile, no obvious sexual differences have been detected when MSL is overexpressed in diploids, illustrating the equilibrium between accumulated histone modifications and some inhibitory factors in genomic balance.

Since the MSL complex plays important regulatory roles in histone modifications during dosage compensation and inverse dosage effects in aneuploids, we compared the global expression levels of MSL2-aneuploids with those aneuploids using normal diploids as controls to investigate the interactions between the MSL complex and genomic imbalance on global gene expression.

It is found that the majority of the X linked genes do not show any increased expression levels even with the H4K16Ac modification levels present on the X chromosome, especially in females. It is illustrated that the de novo MSL complex assembly on the X chromosomes does not directly induce any increased expression of X chromosome genes in these unbalanced trisomic female genomes, which is coincident with the previously published results in normal diploid genomes^40^. On the contrary, it was found that the expression of X chromosome genes is further decreased on the basis of aneuploidy levels in both MSL2-trisomy 2L and MSL2-metafemales, suggesting that the assembly of the MSL complex includes an inhibitory effect on gene expression on the X chromosome, which counteracts the high levels of histone acetylation. In addition, the gene expression level of the autosomes in aneuploid females also decreased significantly, which may be due to the sequestration of histone modification factors to the X chromosome such as acetyltransferase, which were originally present on all chromosomes, resulting in the decrease of autosomal transcription activation and gene expression. These results are in accordance with the previous data of autosomal gene expression changes in normal diploid using autosomal *mini-white* reporter genes based on northern blots^40^. All the above data illustrate that the MSL complex mediates a stronger inhibitory function on gene expression by modulating histone modifications in genomic imbalance. Studies have shown that the altered regulatory stoichiometry is a major contributor to genomic imbalance, which is probably not related with DNA methylation based on the global analyses in different aneuploids^9^. And some other results illustrated an increased DNA methylation has been found in human trisomy aneuploids^53,54^. Generally, it has been believed that DNA methylation and histone modifications are related with dosage compensation in animals^55,56^. But how the genomic imbalance influences the MSL complex to mediate histone modifications or any other epigenetic modifications and affect the global gene expression across the unbalanced genomes in details is still unclear.

To explore these interactions, functions associated with histone modifications and chromatin epigenetic modifications in each contrast are also enrichment analyzed with the differentially expressed genes (Fig. S3, C and D). It is found that different histone modifications, including acetylation, methylation and so on, are involved in distinct responses in these unbalanced genomes when ectopically assembled MSL complex (Fig. S3C), consistent with its function to override a high level of histone modifications^40^. And similar diverse regulation of chromatin organization was also discovered in modulating the genomic imbalance (Fig. S3D), coincident with the function of a chromatin remodeler, ISWI, during dosage compensation^57^. All these results indicate that the MSL complex might affect the global expression of genomic imbalance through post-translational modifications of core histones and altering the dynamic status of chromatin compaction.

In the previous studies, sexual dimorphism has been demonstrated in unbalanced genomes^34^. To further investigate how the MSL complex mediates global expression in genomic imbalance between different sexes, we found that the overexpression of MSL2 has more critical effects in trisomic 2L females and metafemales than in trisomy 2L males, which is probably because MSL2 initiates the assembly of the MSL complex in females, but in males the endogenous MSL complex is already present. More interestingly, there is no obvious changes of X chromosome genes when MSL is de novo recruited by over-expressed MSL2 protein in normal diploid females and males^40^. It is also further confirmed that the interactions between the effect of aneuploidy and the MSL complex would result in the changes of global gene expression and sexual dimorphism, and this result is also easily distinguished based on gene coexpression analyses (Fig. 4; Fig. S2). Also, the expression of genes on the altered dosage of chromosomes was also decreased to an extent when de novo MSL complex is over-expressed in these unbalanced genomes, which further proves that the MSL complex has some dynamic functions of coordinating the global expression across the genome.

In addition, sex-biased genes showed a stronger response to MSL2 overexpression than non-sex-biased genes in aneuploid females including 2L trisomy females and metafemales, in which the ratio of non-sex-biased genes is still close to 1 meaning no change. However, both the female and male biased genes showed significant decreases, especially the male biased genes, with the peak of ratio dropping to nearly 0.67, which seems to be similar to the inverse dosage effect of aneuploidy. In order to further identify the reasons, similar analyses of the overexpression of MSL2 in normal diploid *Drosophila* are also conducted, and in MSL2-diploid only a minor decrease of female-biased genes in female *Drosophila* is found rather than any expression changes of male-biased genes and non-sex-biased genes (Fig. S5). Therefore, it is concluded that the overexpression of MSL2 could produce regulatory effects on sex-biased genes and induce sexual dimorphism in unbalanced genomes, but not in a balanced situation, indicating sex-related interactions between the MSL complex and the inverse dosage effect in aneuploidy as well.

To further determine their intersection, the ratio distributions of several genes associated with different functional categories, including mitochondria, ribosome, signal transduction and transcription factor, are also analyzed when comparing MSL2-aneuploid with relative aneuploidy based on the controls of normal diploids (Fig. S6). It is found that most of the genes exhibit similar ratio distributions as the genome changes in both autosomal and the X chromosome aneuploids. However, the expression level of mitochondria associated genes in MSL2-trisomy 2L aneuploidy has been brought back to the level of wild type in females, in which the ratio distribution is around 1.0 (Fig. S6A), indicating the MSL complex relieves the inverse dosage effect in these unbalanced situations. In contrast, there is no changes identified in males (Fig. S6, E, F, G and H), which is a similar trend as its distribution in MSL-metafemale (Fig. S6, I, J, K and L). With regard to the ribosome genes, all the distribution lines have no changes in both unbalanced genomes (Fig. S6, B, F and J), representing no further effect on gene expression when the MSL complex is added. The sexual dimorphism and the X chromosome specific response to genomic imbalance are also illustrated according to the changes of these different categories.

Based on the differential analyses, the inverse dosage effect showed much broader influences in 2L trisomy than in XXX metafemales (Fig. S3A). When comparing the sexual differences in 2L trisomy, it is concluded that the inverse dosage effect did induce much more differentiated genes in males than in females. Two copies of X chromosomes in diploid females are equal to the copy number of autosomes, which is a balanced genome. In contrast, the X chromosome in males showed an unbalanced situation, albeit evolved to survive, in which all the other chromosomes are two copies (Fig. S1E). However, when the MSL complex is assembled in these unbalanced genomes, it produces much more differentially expressed genes in female aneuploids than in male (Fig. S3C), coincident with the above results of global expressions, indicating that these sexual dimorphisms are contributed by the endogenous MSL complex and inverse dosage effect.

Meanwhile, a large number of uniquely differentially expressed genes in females and males have been identified, providing some possible regulated candidate genes in the sex dimorphism of the aneuploidy effect.

In order to find the possible responsive molecules that the MSL complex interacts with aneuploid and explore the possible mechanisms, we intersected the aneuploid responsive genes and the aneuploid expression genes affected by MSL2 and analyzed the protein interactions. 12 candidate genes are screened involving in energy metabolism, material metabolism, protein processing and many other aspects. Due to the extremely high sensitivity and resolution of TSA-FISH technology, we observed the subembryonic and subcellular localization patterns of the corresponding transcripts, and conducted a semi-quantitative analysis of the relative expression levels of these genes and some components of the MSL complex. We found that functional proteins such as *Hsc70-4* and *ERp60* in the candidate genes may be important intermediary molecules for MSL complex. In particular, we found that their relative expression levels in all stages of embryonic development have a declining trend after the overexpression of MSL2 although these candidate genes are located in different chromosomes. This also confirmed that the MSL complex may have a negative regulatory trans-effect on gene expression rather than directly up-regulating gene expression level at the cellular level.

## Methods

### *Drosophila* stocks and crosses

Four different cross combinations were included in this study. The cross method is shown in Fig. S1. Trisomy chromosome 2 left arm (2L) larval samples were obtained from crosses of y; C(2L)dp; F(2R) bw females with Canton S males. Trisomy 2L with ectopic MSL2 expression larval samples were obtained from crosses of y; C(2L)dp; F(2R) bw females with males, which have homozygous MSL2 transgenes on chromosome 2R. In these two kinds of crosses, monosomy 2L will lead to early lethality and only trisomy larvae can survive to the third instar stage. Metafemale (XXX) larval samples were obtained from crosses of C(1)DX, ywf/winscy females with Canton S males. Metafemales with ectopic MSL2 expression larval samples were obtained from crosses of C(1)DX, ywf/winscy females with males, which have homozygous MSL2 transgenes on chromosome 2R. In these two kinds of crosses, the phenotype of y + can be used to screen for trisomy X larvae. The normal diploid larval samples were collected from the Canton S stock. The MSL2 transgene strains were constructed in a previous study^40^. Mutations, genes, and chromosomal balancers are described in Flybase (http://flybase.org).

### RNA-Seq library construction and sequencing

The whole third instar larvae were used for sequencing, with about 20 larvae per sample. The samples were sequenced in four groups separately. RNA isolation, library preparation, and sequencing were performed as described previously^40^. For trisomy 2L group (trisomy 2L female and male, diploid female and male, each genotype has four biological replicates) and metafemale group (metafemale, diploid female and male, each genotype has three biological replicates), single-end 50 bp protocol with Illumina HiSeq 2000 sequencers were performed. For MSL2-trisomy 2L group (MSL2-trisomy 2L female and male, trisomy 2L female and male, each genotype has three biological replicates) and MSL2-metafemale group (MSL2-metafemale and metafemale, each genotype has two biological replicates), the samples were sequenced with Illumina HiSeq X-ten sequencers using the paired-end 150 bp protocol.

### RNA sequencing analyses

Data output from Illumina RNA sequencing was processed based on the HISAT2-StringTie pipeline^58^. Briefly, RNA-Seq reads of each sample were mapped to the NCBI *Drosophila melanogaster* genome (GCF_000001215.4_Release_6_plus_ISO1_MT_genomic.fna) in conjunction with the corresponding annotation (GCF_000001215.4_Release_6_plus_ISO1_MT_genomic.gff) using HISAT2 (version 2.1.0) with the default parameters^59^. Subsequently, SAMtools^60^ was used to convert the alignment files from SAM format to BAM format. Transcripts were assembled and quantitated using StringTie (version 1.2.2)^61^, and the raw read counts information were extracted using the Python script prepDE.py (https://ccb.jhu.edu/software/stringtie/dl/prepDE.py). The dataset of diploid *Drosophila* with ectopic MSL2 expression was obtained from the GEO database (GSE41570)^40^.

### Relative quantitative PCR

The third instar larvae were used for RNA isolation and RT-qPCR. RNA was isolated using TRIzol Reagent from Invitrogen. cDNA was synthesized using the TransScript one-step gDNA Removal and cDNA Synthesis SuperMix (TransGen Biotech). *β-tubulin* gene was the internal control, using primers 5′-AGCTCAGCACCCTCTGTGTAAT-3′ and 5′-AGCTGGAGCGCATCAATGTGTA-3’ to generate a 208-bp product. The real-time PCR reactions were processed using diluted cDNA (200 ng) with the TransScript Tip Green qPCR SuperMix (+ Dye II) (TransGen Biotech). Three biological and technical repeats were applied to each pair of primers. The relative quantification was measured based on the ΔCt analysis from ABI Quant Studio 6 Flex and using QuantStudio Real Time PCR Software v1.3 (Applied BioSystems). The primers used in relative quantitative PCR are listed in Table S1.

### Ratio distribution

A ratio distribution analysis can visualize the trends of expression changes between experimental material and the control, which could be subtle, and show the entire landscape of the modified effects. Specifically, read counts were normalized by dividing them by the total number of mapped reads in the replicate to calculate CPM (counts per million). Because the trisomic samples and the MSL2-trisomic samples are from two sequencing batches, Combat function from R package sva (version 3.36.0) was performed to remove batch effects^62^. Here a temporary logarithmic transformation was performed so that the data is more suitable for adjustment. Then the CPM values not greater than 0 were removed, so that the analysis will not be disturbed by these lowly expressed transcripts which would generate ratios of extreme values. Normalized counts were averaged across biological replicates, followed by calculating the ratio of each transcript by dividing the mean of experimental values by the control values. Ratio distribution plots were generated using ggplot2 package^63^ in the R program^64^.

### Weighted gene coexpression network analysis (WGCNA)

Gene co-expression networks were generated using the R package WGCNA (version 1.69)^65^. The logarithmic CPM of the 10,000 genes with the highest average expression were analyzed in autosomal aneuploidy group and sex chromosomal aneuploidy group, respectively. Briefly, the Pearson correlation coefficients were calculated in selected samples. The correlation matrix was transformed using a threshold power (11 in autosomal aneuploidy group and 17 in sex chromosomal aneuploidy group), and then converted into Topological Overlap Matrix (TOM). Gene modules were defined using dynamic hybrid tree cut method on the cluster dendrogram of distance matrx in one block. This approach defined six and five signed gene co-expression modules in our two datasets. The expression of each module was summarized as a module eigengene in every sample. Gene ontology enrichment analysis was conducted using R package clusterProfiler (version 3.16.1)^66^. The gene lists of transcription factors and transcription cofactors were obtained from AnimalTFDB 3.0^67^. Heatmaps were generated using the R package ComplexHeatmap^68^.

### Differential expression analysis

Differential gene expression analysis was performed using DESeq2 (version 1.28.1)^69^. Here we conducted separate tests in each sex and each batch, because of the inherent differences in dispersion between sexes and the systematic differences between batches, and these differences should not be conflated. A pre-filtering was performed to keep only rows that have at least 10 reads total. An appropriate local fit was used by providing the argument fitType = "local" because the parametric curve does not fit the dispersion trend well in some of our datasets. The GO annotations were provided by org.Dm.eg.db (version 3.11.4). Protein–protein interaction network analysis was conducted using online tool STRING 11.0 (https://string-db.org/)^70^. The networks were further processed by Cytoscape (version 3.7.1)^71^.

### Salivary gland chromosomes immunostaining

Salivary glands from third-instar larvae were dissected and fixed in 3.7% formaldehyde for 1 min. Then the solution was replaced by a mixture of 50% acetic acid and 3.7% formaldehyde to dissociated for 5 min. Chromosomes were probed with anti-SXL (Developmental Studies Hybridoma Bank, M18-s), anti-MSL2 (Santa Cruz, sc-32459), anti-MOF (Santa Cruz, sc-22351) and anti-H4K16Ac (EMD Millipore, 07–329) antibodies at a dilution of 1:100, and probed with an appropriate conjugated secondary antibody (Alexa Fluor 488 and Alexa Fluor 594, Jackson Immuno Research) at a 1:200 dilution. The slides were observed by fluorescence microscope.

### Embryo TSA-FISH

Probe production: Designed primers were used to amplify cDNA templates, which were obtained from reverse transcription of RNA from male larvae. The primers containing flanking T7 promoter elements are listed in Table S3. DNA fragments were concentrated by ethanol precipitation and resuspended in nuclease-free water. Subsequently, antisense RNA probes labeled with digoxigenin (DIG) were synthesized with the DNA fragments using T7 RNA polymerase. The efficiency and accuracy of all PCR amplifications and transcription reactions was systematically verified by agarose gel electrophoresis after each step.

Embryo collection and fixation: All flies were cultured on cornmeal medium at 25 °C. When the embryos are collected, 100 males and 200 females were transferred to homemade cages and cultured on agar juice medium with yeast extract. Embryos were collected based on development stages and processed for fixation and storage as described by Le´cuyer et al.^72^. The collected embryos were dechorionated in a chlorine bleach solution, followed by rinsing the embryos immediately and thoroughly with flowing room temperature tap water to remove residual bleach. Embryos were transfered to a 1.5 mL tube containing 600 μL heptane and 200 µL 4% formaldehyde and shaked for 20 min. Then the embryos were transfered to a new tube with 200 μL heptane and 200 µL methanol and shaked vigorously for 15 s twice followed by rinsing three times with methanol. Finally, all of the liquid were removed and the embryos were stored in methanol at − 20 °C.

Fluorescent in situ hybridization (FISH): FISH using tyramide signal amplification (TSA) in *Drosophila* embryos were performed as described by Jandura et al.^73^. The embryos were permeabilized using proteinase K and post-fixed in 4% PFA for 20 min. After pre-hybridizing in denatured hybridization solution for 3 h at 56 °C, denatured gene-specific probes were added to each sample and hybridize at 56 °C for 16–18 h. DIG-labeled probes were detected using biotin-conjugated mouse monoclonal anti-DIG antibody (1:400), and streptavidin-HRP solution (1:1000). DNA was detected using DAPI solution (1 μg/mL). Antibody detection used cyanine 3-conjugated tyramide diluting 1:80 in activation buffer containing 0.006% of H_2_O_2_. Anti-fade mounting media was added to the samples for the production of microscope slides.

Sample imaging procedure: All images were acquired with a Zeiss LSM880 laser confocal fluorescence microscope using ZEN software. For each sample, a combination of low (20 ×) and high magnification (63 × oil) images were captured. The same parameters were used for these images to make sure the fluorescence intensity analysis reasonable, and the images were analyzed in their original format using ZEN software. Tyramide-Cy3 and DAPI images were pseudo-colored in red and green respectively, using ImageJ, as this color scheme was found to provide the best contrast. Each layer of the Z-stack images was overlaid to obtain the maximum intensity single layer pictures for display.

## Supplementary Information

Supplementary Information.

## Data Availability

The datasets generated during the current study are available in the Gene Expression Omnibus (GEO) database (accession no. GSE162951) and the raw RNA sequencing output data have been deposited in the Sequence Read Archive (SRA) (accession no. SRP296902). The public data used in this study were downloaded from GSE46354, GSE41679 and GSE41570.
