# Supplementary material for: Interaction of Male Specific Lethal complex and genomic imbalance on global gene expression in Drosophila
Source: Sci Rep. 2021 Oct 4;11:19679. doi: 10.1038/s41598-021-99268-y (PMC8490464; doi:10.1038/s41598-021-99268-y)
Supplement: Supplementary file 1 — Supplementary Information. [file 41598_2021_99268_MOESM1_ESM.pdf]

Supplementary Information

Interaction of Male Specific Lethal complex and genomic imbalance on global gene expression in *Drosophila*

Shuai Zhang, Haizhu Qi, Cheng Huang, Lijia Yuan, Ludan Zhang, Ruixue Wang, Yu Tian and Lin Sun

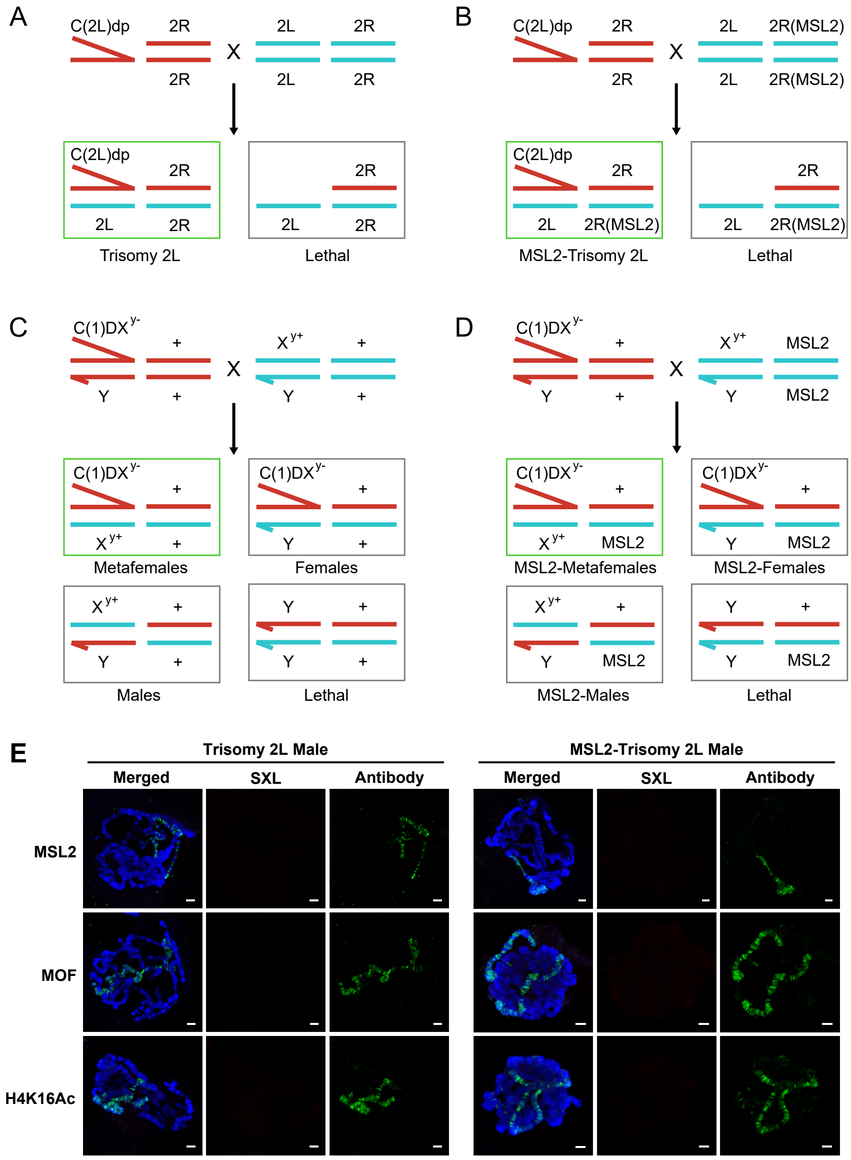

Figure S1. Genetic crosses to generate aneuploidy *Drosophila*. (**A**) Cross of y; C(2L)dp; F(2R) bw females with Canton S males to produce trisomy 2L larvae. (**B**) Cross of y; C(2L)dp; F(2R) bw females with MSL2/MSL2 males to produce MSL2-trisomy 2L larvae. (**C**) Cross of C(1)DX, ywf/winscy females with Canton S males to obtain metafemale larvae. (**D**) Cross of C(1)DX, ywf/winscy females with MSL2/MSL2 males to obtain MSL2-metafemale larvae. (**A**-**D**) The selected offspring genotypes are shown in the green boxes. (**E**) Immunofluorescence of *Drosophila* polytene chromosomes from third instar larvae of trisomy 2L males and MSL2-trisomy 2L males. The red channel is the signal from SXL and the green channel is the signal from antibodies of MSL complex components. DNA is stained with DAPI in blue. Scale bars, 5 µm.

Table S1. Primers used in RT-PCR

Genes	Chromosome	Left primer	Right primer
Karl	X	GAGAAGCTCGTGGGCAACAT	GTGTCGTAGTCCGTGTCCAGAA
CG9577	X	CGTCGCCGTAAAGACAACCA	CACAGCCTGGGCGAAGTC
CG15771	X	TCTATGCCGCCTGCAACTTT	TGCCACGTCTTCTGGAAACC
HERC2	X	TCCAGCAACTGCTTGAACTG	TGTTCAGCTGACCGAGTACG
Myb	X	TTGAAATGCGGTCCGATAAT	GGGACAGAACAAAAGCGGTA
Tlk	X	TCTCCTCTTTGCGGTTCATT	CATATGATGTTGATTTCGGGG
jet	2L	GAACTCCACCAGCTTGCTTT	AACGAATGCGTCAACATCAC
CG43773	2L	GAGGAGTCCTGTACTATGCAAATC	CAGCGACTGGTACAAATGTAGT
Aac11	2L	GAGCAGGCGGAGCTAAATAA	TCATGTTCCAGGTCTTGATGG
magu	2R	TACATCTGCGTCCAAAGGC	GAATCCGAGAGAGATGGCTG
CG33468	2R	CTGGAAAGGTATTTGCGAAGTG	CCTGATCTGTTGTCGCATAGT
CG42694	2R	GGCTCCGATATCAAACCAATCT	GCTTACTCCAAGTTGCACAAAC
CG4716	2R	GAGAGCACCGGAATCCATATT	CAGTGTGCTCCGTAGTTGTT
Kap-α1	3L	TTGACCTGCATTAGCCACCT	CATGATAACCATCAGCTGCG
CG16758	3L	GGTATTCGCCTTCAGCCTCAT	GTTCGCAGCAGGCCTTTTG
LysB	3L	CCCTCCTAACCGACGACATC	CCGCTGCAGTACTTCCATGTG
Sp7	3R	AGCACACGAATGGCAATCAA	GATCCAATCGCTGCTTTATGG
Hsp70	3R	GCTTGATTGGTCGCAAGTTC	CGGTCTCCTTCATCTTGGTAAG

Table S2. Validation of RNA-sequencing using relative quantitative PCR

Genes	Location	MSL2-Metafemale/ Metafemale		MSL2-Trisomy 2L Female/Trisomy2L Female		MSL2-Trisomy 2L Male/Trisomy2L Male	
		RNA-seq	RT-PCR	RNA-seq	RT-PCR	RNA-seq	RT-PCR
Karl	X	0.821	0.937	0.883	0.882	0.952	0.761
CG9577	X	0.565	0.564	0.410	0.122	0.847	0.727
CG15771	X	0.680	0.776	1.051	1.212	0.722	0.716
HERC2	X	0.371	0.399	1.393	1.240	1.465	1.236
Myb	X	0.823	1.223	0.724	0.397	0.795	0.760
Tlk	X	0.216	0.389	0.652	1.222	0.897	1.231
jet	2L	0.897	0.644	0.776	0.879	0.774	0.887
CG43773	2L	0.425	0.763	0.653	0.342	0.563	0.235
Aac11	2L	0.633	1.002	0.986	1.320	1.232	0.653
magu	2R	0.583	0.343	0.871	1.453	0.752	1.043
CG33468	2R	0.032	0.074	0.064	0.061	0.06	0.095
CG42694	2R	0.112	0.150	1.743	1.970	0.798	0.715
CG4716	2R	1.532	0.989	0.384	0.309	0.365	0.276
Kap-α1	3L	0.689	1.002	0.796	0.689	0.774	0.506
CG16758	3L	0.812	0.548	0.600	0.667	1.340	1.607
LysB	3L	1.303	1.437	0.271	0.491	0.456	0.468
Sp7	3R	0.914	0.942	0.637	0.606	0.611	0.670
Hsp70	3R	1.021	0.883	2.503	2.741	1.635	0.986

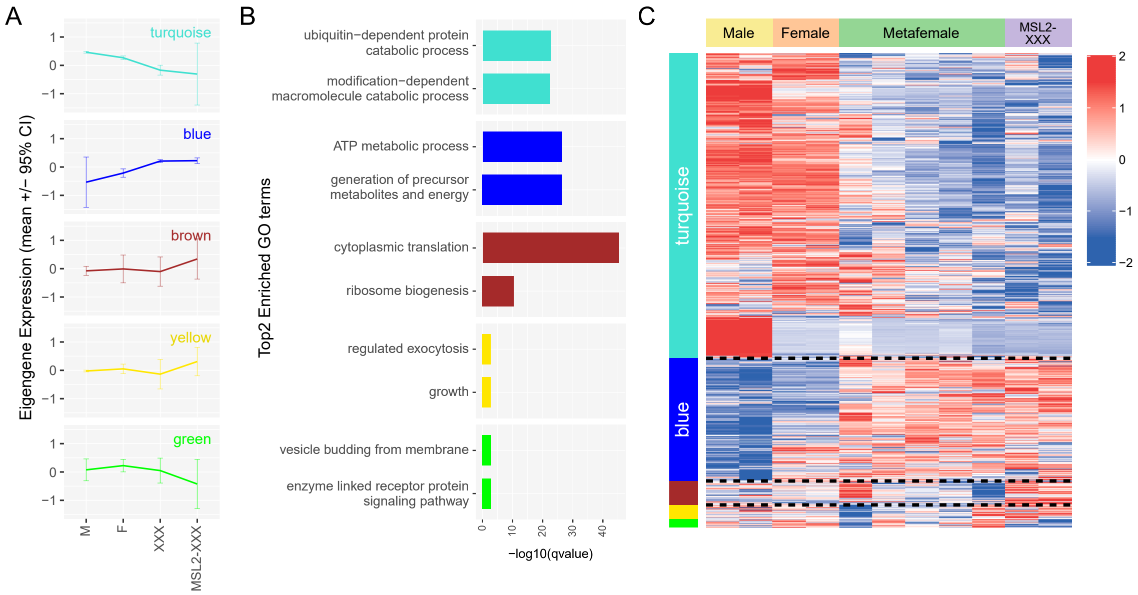

Figure S2. Weighted gene coexpression network analysis (WGCNA) of sex chromosomal aneuploidy *Drosophila*. (**A**) Dot and line plots showing the eigengene expression (mean +/- 95% confidence interval) of each gene-coexpression module by genotypes in sex chromosomal aneuploidy groups. The modules are ordered according to the number of genes they contain. M, male; F, female; XXX, metafemale; MSL2-XXX, MSL2-metafemale. (**B**) Top two GO term enrichments for each module. (**C**) Heatmap showing the expression level of genes in every coexpression module. Each column is a biological repetition and each row is a gene. Hierarchical clustering is carried out only within the module and genotype groups. The horizontal dotted lines divide the three largest modules. The 10,000 genes with the highest average expression and relatively low variation within groups were involved in the coexpression network analysis. Gray modules are not considered in the plots.

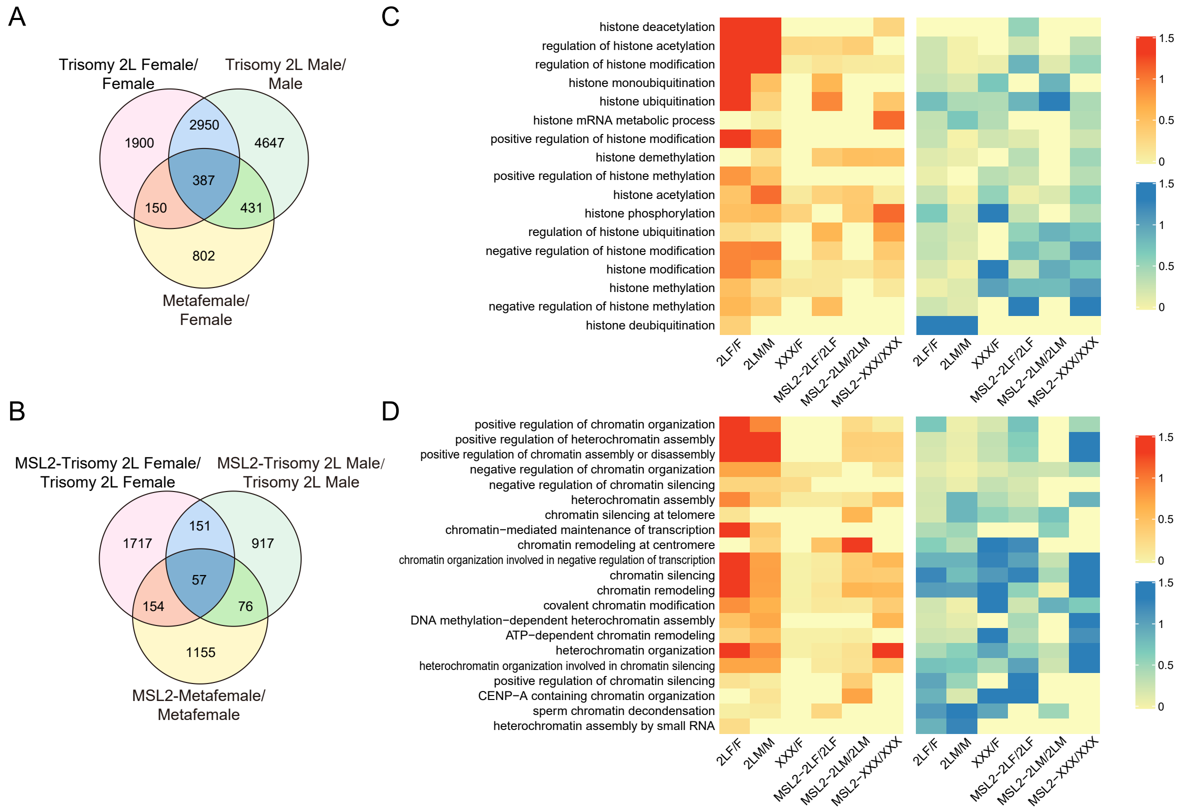

Figure S3. Differentially expressed genes and functional enrichment analysis. (**A** and **B**) Venn diagrams show the number of differentially expressed genes in trisomy/normal diploid (**A**) and MSL2-trisomy/trisomy (**B**). Genes with padj lower than 0.05 are selected as differentially expressed genes. (**C** and **D**) Functional enrichment analysis with the differentially expressed genes in each contrast. (**C**) Enrichment analysis of functions associated with histone modification in each contrast. (**D**) Enrichment analysis of functions associated with chromatin epigenetic modification in each contrast. Up-regulated genes are shown in the left panel and down-regulated genes are shown in the right panel. The legends represent -log10 (p-value). Red saturation indicates the enrichment level of up-regulated genes. Blue saturation indicates the enrichment level of down-regulated genes. The vertical axis is GO terms and the horizontal axis is contrast groups. F, female; 2LF, trisomy 2L female; MSL2-2LF, MSL2-trisomy 2L female; M, male; 2LM, trisomy 2L male; MSL2-2LM, MSL2-trisomy 2L male; XXX, metafemale; MSL2-XXX, MSL2-metafemale.

Table S3. Primers used in FISH

Genes	Chromosome	Left primer	Right primer
Hel25E	2L	TTCACATCGAAACGATCCTG	TTGTGAAGTCTGTGCAACG
RpS2	2L	CATATGTAGCCTTGGCGAAG	ATCTACCTGTACTCGCTTCC
msl-1	2L	GCATCCCTACACTTTCGATC	CCCGTCTGATATTCGGAAAC
mle	2R	AAGGGCGAGACCATAGATAG	CAGTAATCGAAGCTGAGGTG
Mal-A3	2R	CTATTCCACGCTCCTTCTTC	GATCTAAACTACCGCAACCC
TER94	2R	CTTTTGACCCTGATGGATGG	TTAGCCTTCAGGATAGCCTC
ERp60	2R	TAAGGAAGTCGTCTACCTCG	ACTACCAGAAGAACCCCAAG
Vha44	2R	GGCTTATTGAACCACCAGTC	GATCTCAAGGGTAAATCCGC
scf	3L	CACCAAGGATGAGTTTACCG	CGAAAACGTCGTACTTGTCT
Hsc70-4	3R	GGTGCTTACCAAGATGAAGG	CATCGGGATTGATCGACTTG
CG1894	3R	CTGAGGTAACAAAGTCTGGC	CGAAGCTCAAAGAATAGGGG
CR45570	3R	CACCGCTAGAAATTGGACTC	AACGAGGATTGGAGTAGGAG
roX1	X	GTTGGACCGGATTTCATAGG	CTACACCCAGAAGAAACTGC
mof	X	GGCCCTTCCAATACTTGATC	GTCCTATTGCGAGAACAGTC
ND-B16.6	X	GGGCGTAGAACTCCTTGAAA	GCCTGGGCATCTACTACTTG
Ran	X	CACAGAGGACGATTGGTATG	GCTCAGGAAGGTCAGGATAT

The sequences of promoters connected to the flanks of the primers are not listed in the table. The forward primers connect T3 promoter: TGTTGGGAAATCACTCCCAATTAA. The reverse primers connect T7 promoter: GTAATACGACTCACTATAGGGAGACCAC.

Figure S4. Embryo-FISH of candidate genes. The following images represent candidate genes *Hel25E*, *RpS2*, *msl-1*, *mle*, *Mal-A3*, *ERp60*, *Vha44*, *scf*, *Hsc70-4*, *CG1894*, *CR45570*, *roX1*, *mof*, *ND-B16.6*, and *Ran*, respectively. (**A**) Candidate gene expression patterns in entire embryo. The genotype of the sample is shown in the horizontal axis above, and the development stage of the sample is shown in the left vertical axis. The red pseudo-color is the signal from probe and the green pseudo-color is the signal from nucleus. Scale bars, 80 µm. (**B**) RNA subcellular location patterns in wild type embryos. The image shown at the bottom right shows a locally enlarged subcellular localization pattern. Scale bars, 30 µm. (**C**) Relative fluorescence intensity of candidate gene RNA signal in trisomy 2L and MSL2-trisomy 2L compared with normal diploid. (**C1**-**C4**) The four plots represent stage 1-5, stage 6-11, stage 12-13 and stage 14-17 of embryonic development, respectively. The relative fluorescence intensity is determined by comparison with DAPI signal. The expression of wild-type embryos at each stage of development are set at 1. Asterisk denotes a Student's *t* test P-value <0.05.

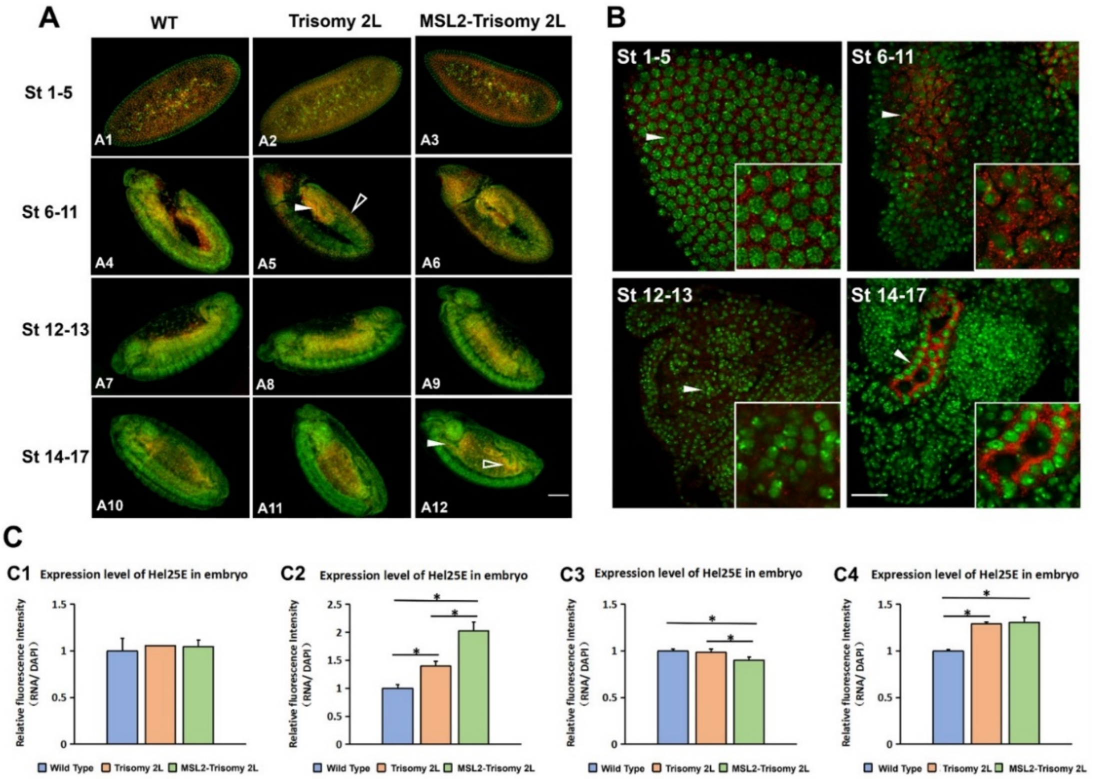

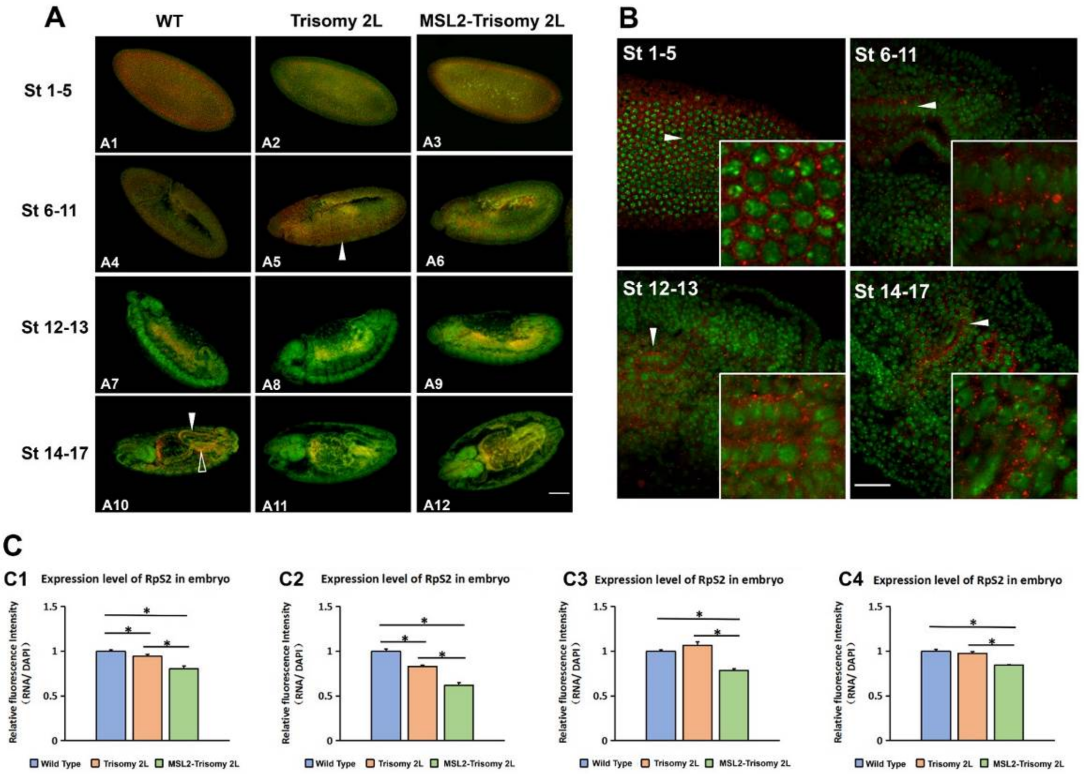
A
WT
Trisomy 2L
MSL2-Trisomy 2L
St 1-5
St 6-11
St 12-13
St 14-17
A1
A2
A3
A4
A5
A6
A7
A8
A9
A10
A11
A12
B
St 1-5
St 6-11
St 12-13
St 14-17
C
C1 Expression level of RpS2 in embryo
C2 Expression level of RpS2 in embryo
C3 Expression level of RpS2 in embryo
C4 Expression level of RpS2 in embryo
Relative fluorescence Intensity (RNA/ DAPI)
Wild Type
Trisomy 2L
MSL2-Trisomy 2L

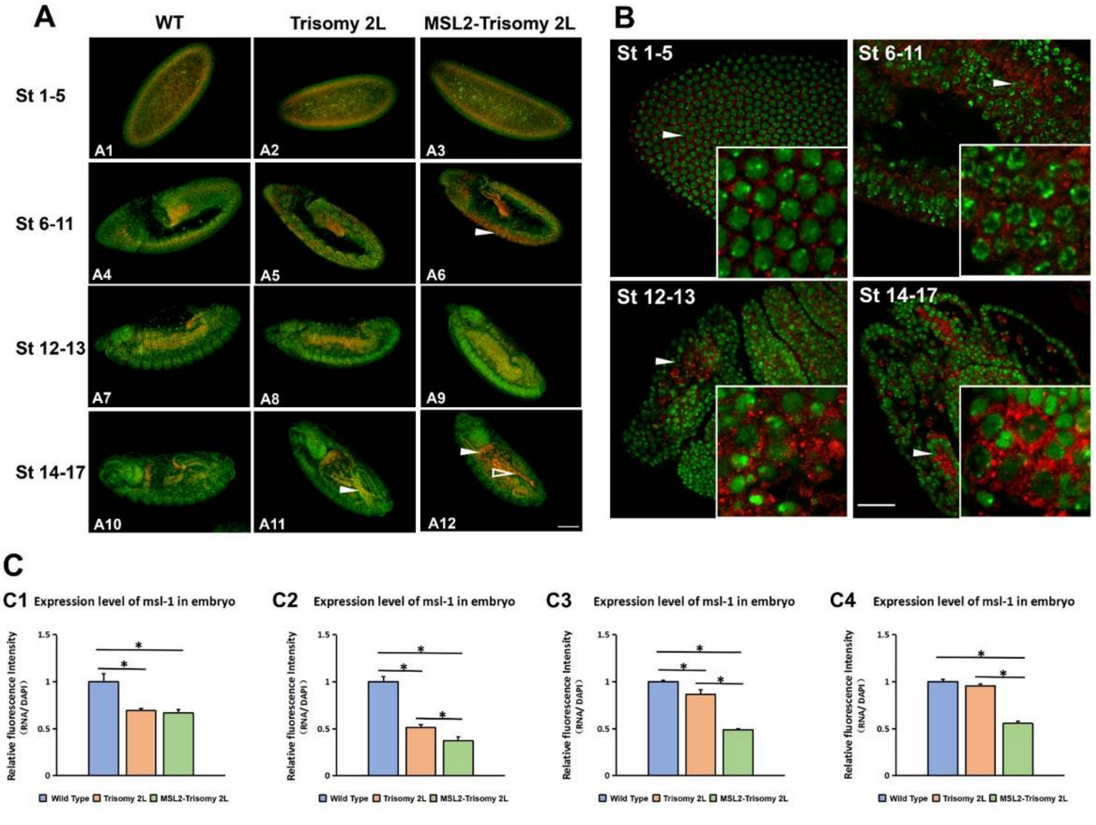
A
WT
Trisomy 2L
MSL2-Trisomy 2L
St 1-5
St 6-11
St 12-13
St 14-17
A1
A2
A3
A4
A5
A6
A7
A8
A9
A10
A11
A12
B
St 1-5
St 6-11
St 12-13
St 14-17
C
C1 Expression level of msl-1 in embryo
C2 Expression level of msl-1 in embryo
C3 Expression level of msl-1 in embryo
C4 Expression level of msl-1 in embryo
Relative fluorescence Intensity (RNA/ DAPI)
Wild Type
Trisomy 2L
MSL2-Trisomy 2L

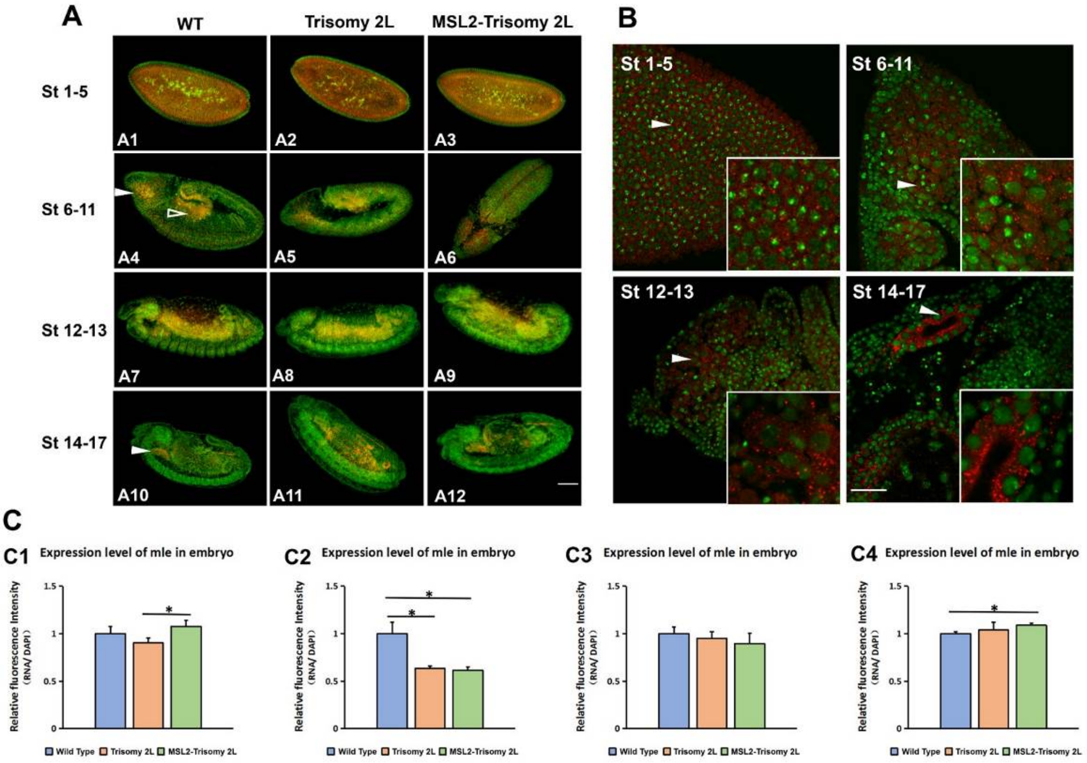
A
WT
Trisomy 2L
MSL2-Trisomy 2L
St 1-5
St 6-11
St 12-13
St 14-17
A1
A2
A3
A4
A5
A6
A7
A8
A9
A10
A11
A12
B
St 1-5
St 6-11
St 12-13
St 14-17
C
C1 Expression level of mle in embryo
C2 Expression level of mle in embryo
C3 Expression level of mle in embryo
C4 Expression level of mle in embryo
Relative fluorescence Intensity (RNA/ DAPI)
Wild Type
Trisomy 2L
MSL2-Trisomy 2L

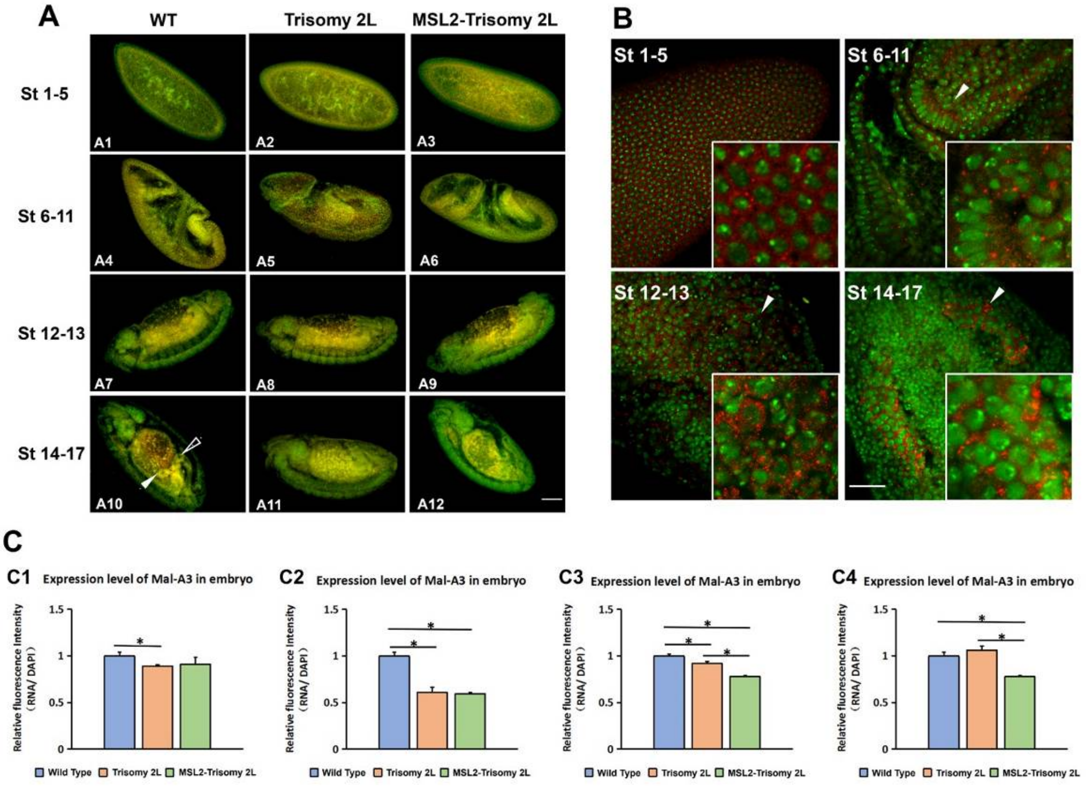
A
WT
Trisomy 2L
MSL2-Trisomy 2L
St 1-5
St 6-11
St 12-13
St 14-17
A1
A2
A3
A4
A5
A6
A7
A8
A9
A10
A11
A12
B
St 1-5
St 6-11
St 12-13
St 14-17
C
C1 Expression level of Mal-A3 in embryo
C2 Expression level of Mal-A3 in embryo
C3 Expression level of Mal-A3 in embryo
C4 Expression level of Mal-A3 in embryo
Relative fluorescence Intensity (RNA/ DAPI)
Wild Type
Trisomy 2L
MSL2-Trisomy 2L

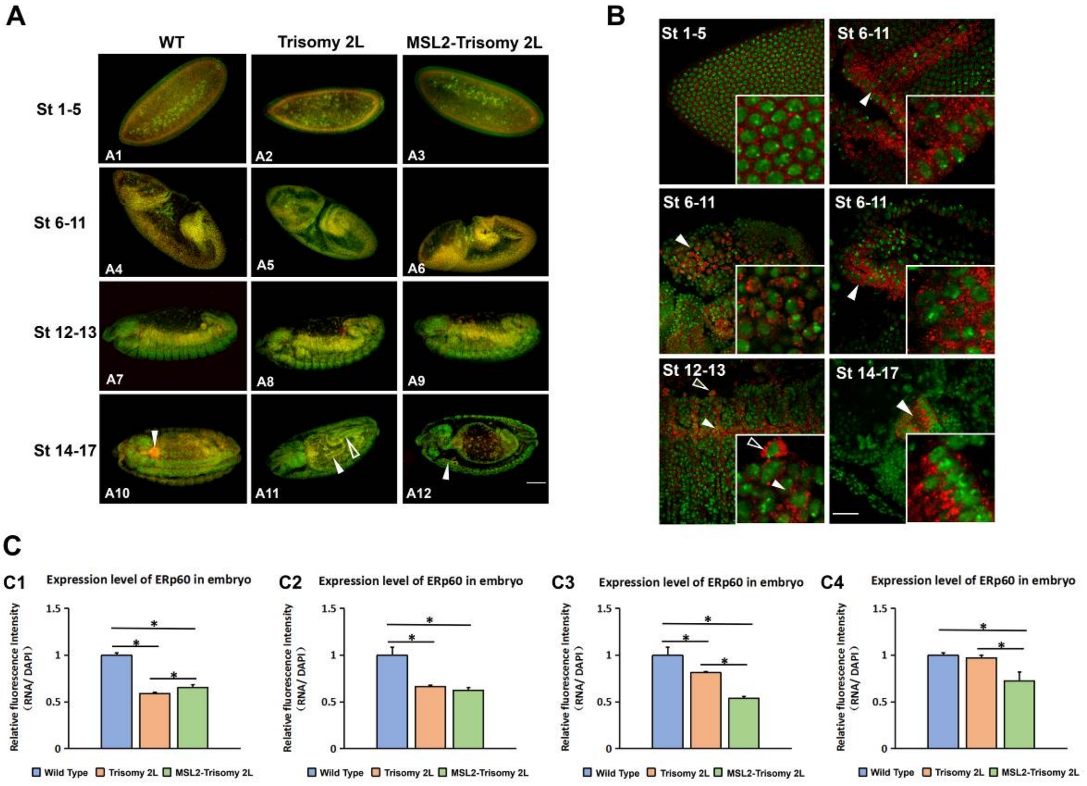
A
WT
Trisomy 2L
MSL2-Trisomy 2L
St 1-5
St 6-11
St 12-13
St 14-17
A1
A2
A3
A4
A5
A6
A7
A8
A9
A10
A11
A12
B
St 1-5
St 6-11
St 6-11
St 6-11
St 12-13
St 14-17
C
C1
Expression level of ERp60 in embryo
C2
Expression level of ERp60 in embryo
C3
Expression level of ERp60 in embryo
C4
Expression level of ERp60 in embryo
Relative fluorescence intensity (RNA/ DAPI)
Wild Type
Trisomy 2L
MSL2-Trisomy 2L

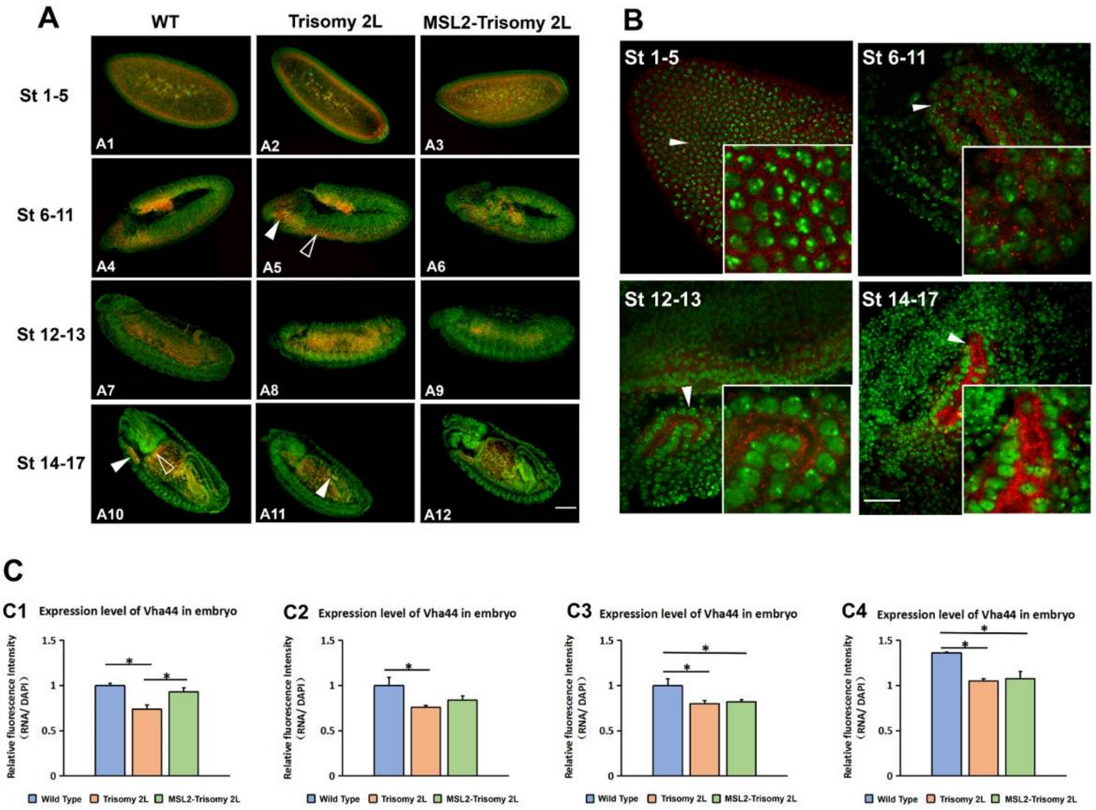
A
WT
Trisomy 2L
MSL2-Trisomy 2L
St 1-5
St 6-11
St 12-13
St 14-17
A1
A2
A3
A4
A5
A6
A7
A8
A9
A10
A11
A12
B
St 1-5
St 6-11
St 12-13
St 14-17
C
C1
Expression level of Vha44 in embryo
C2
Expression level of Vha44 in embryo
C3
Expression level of Vha44 in embryo
C4
Expression level of Vha44 in embryo
Relative fluorescence intensity (RNA/ DAPI)
Wild Type
Trisomy 2L
MSL2-Trisomy 2L

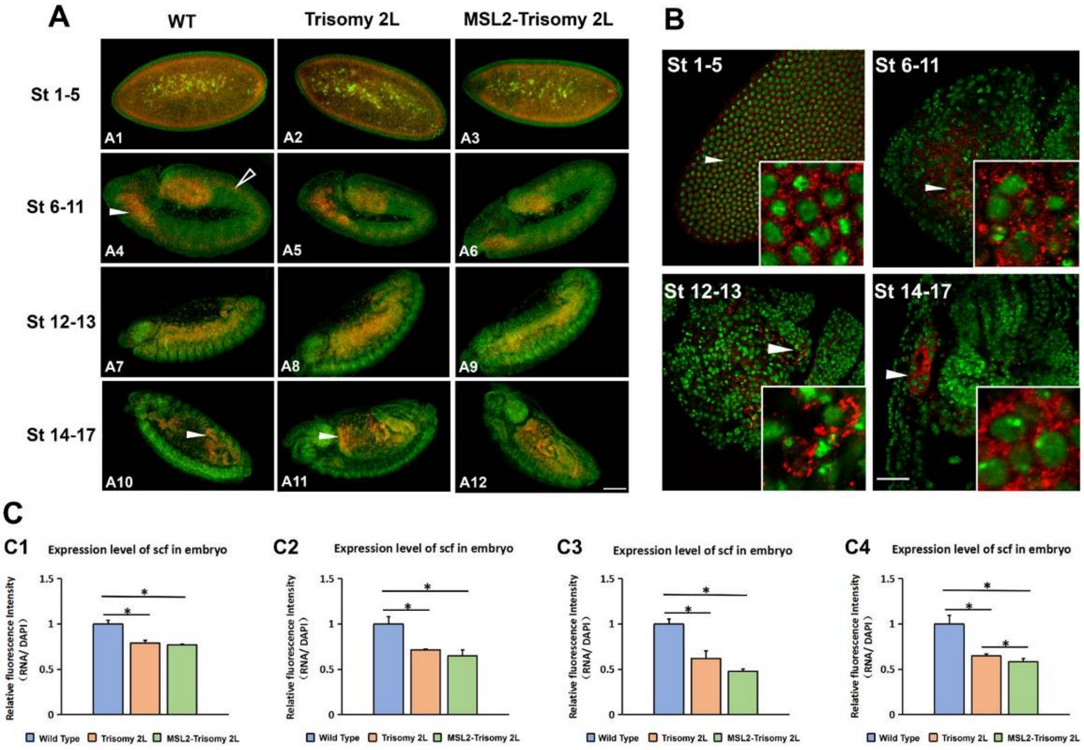
A
WT
Trisomy 2L
MSL2-Trisomy 2L
St 1-5
St 6-11
St 12-13
St 14-17
A1
A2
A3
A4
A5
A6
A7
A8
A9
A10
A11
A12
B
St 1-5
St 6-11
St 12-13
St 14-17
C
C1 Expression level of scf in embryo
C2 Expression level of scf in embryo
C3 Expression level of scf in embryo
C4 Expression level of scf in embryo
Relative fluorescence Intensity (RNA/ DAPI)
Wild Type
Trisomy 2L
MSL2-Trisomy 2L

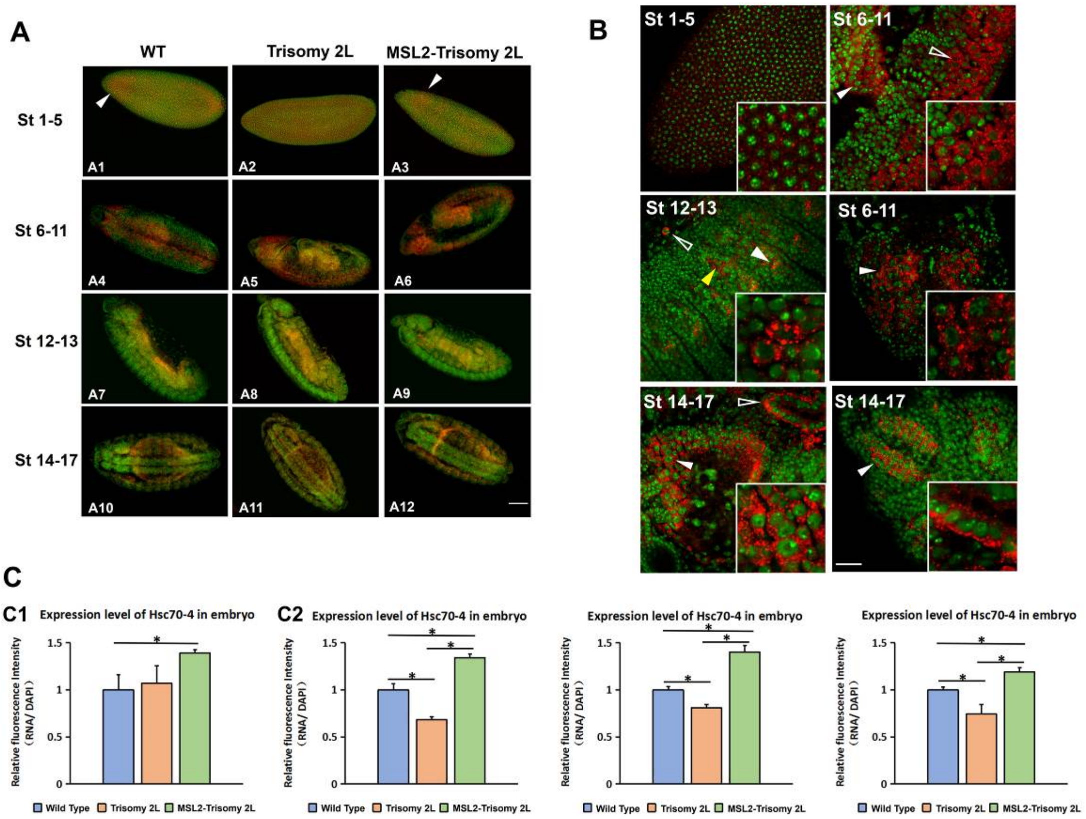
A
WT
Trisomy 2L
MSL2-Trisomy 2L
St 1-5
St 6-11
St 12-13
St 14-17
A1
A2
A3
A4
A5
A6
A7
A8
A9
A10
A11
A12
B
St 1-5
St 6-11
St 12-13
St 6-11
St 14-17
St 14-17
C
C1 Expression level of Hsc70-4 in embryo
C2 Expression level of Hsc70-4 in embryo
Expression level of Hsc70-4 in embryo
Expression level of Hsc70-4 in embryo
Relative fluorescence Intensity (RNA/ DAPI)
Wild Type
Trisomy 2L
MSL2-Trisomy 2L

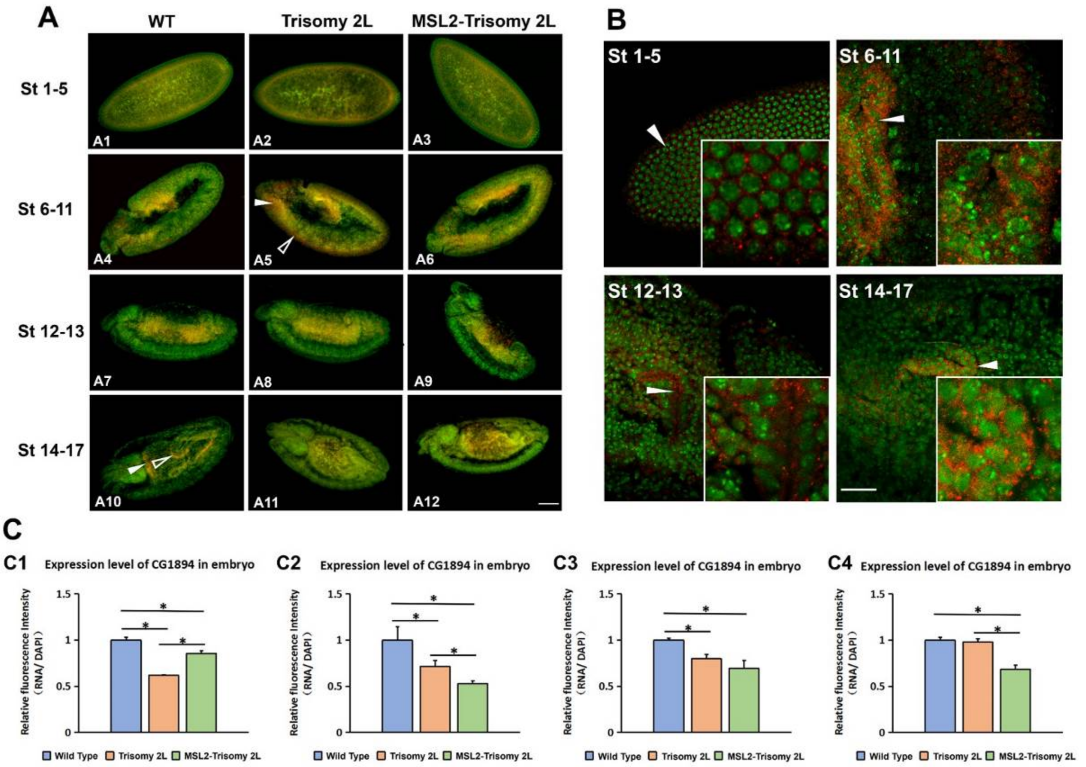
A
WT
Trisomy 2L
MSL2-Trisomy 2L
St 1-5
St 6-11
St 12-13
St 14-17
A1
A2
A3
A4
A5
A6
A7
A8
A9
A10
A11
A12
B
St 1-5
St 6-11
St 12-13
St 14-17
C
C1 Expression level of CG1894 in embryo
C2 Expression level of CG1894 in embryo
C3 Expression level of CG1894 in embryo
C4 Expression level of CG1894 in embryo
Relative fluorescence Intensity (RNA/ DAPI)
Wild Type
Trisomy 2L
MSL2-Trisomy 2L

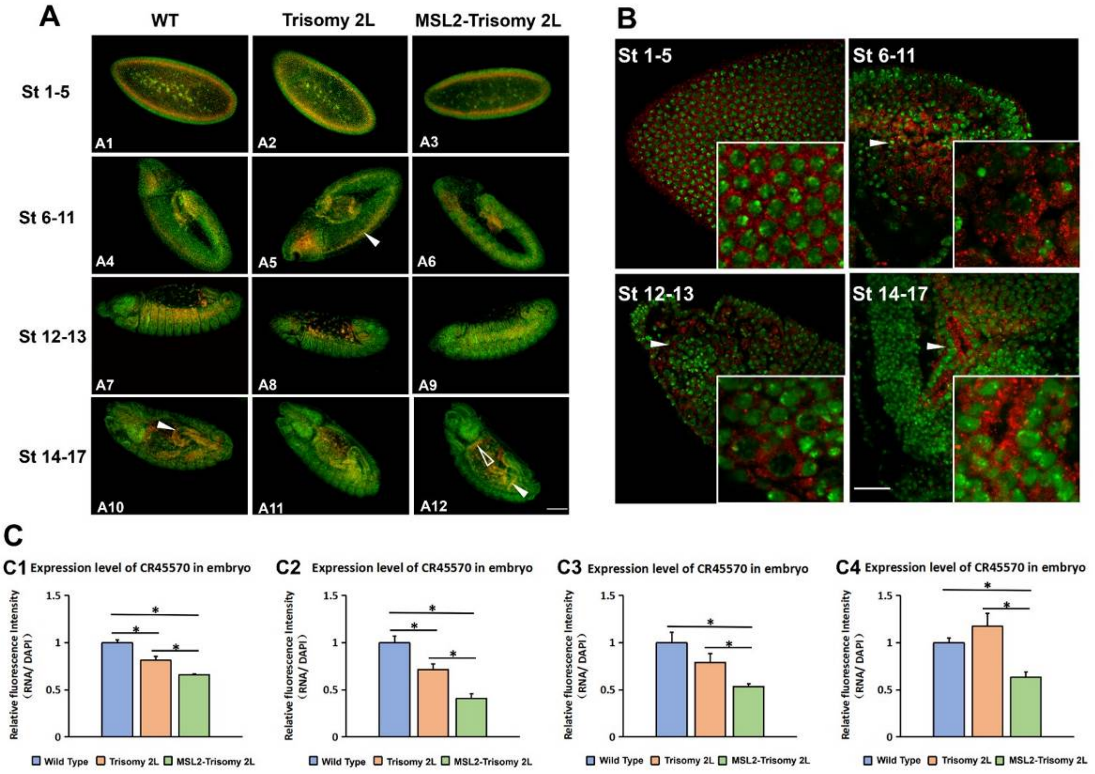
A
WT
Trisomy 2L
MSL2-Trisomy 2L
St 1-5
St 6-11
St 12-13
St 14-17
A1
A2
A3
A4
A5
A6
A7
A8
A9
A10
A11
A12
B
St 1-5
St 6-11
St 12-13
St 14-17
C
C1 Expression level of CR45570 in embryo
C2 Expression level of CR45570 in embryo
C3 Expression level of CR45570 in embryo
C4 Expression level of CR45570 in embryo
Relative fluorescence Intensity (RNA/ DAPI)
Wild Type
Trisomy 2L
MSL2-Trisomy 2L

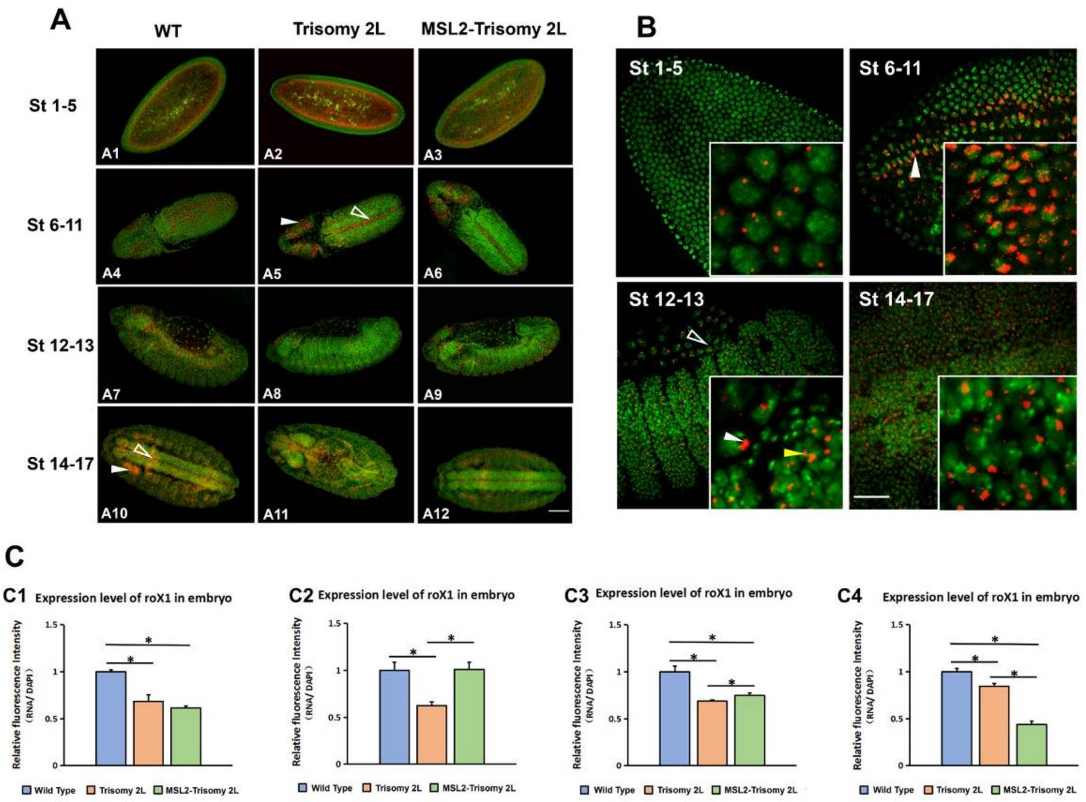
A
WT
Trisomy 2L
MSL2-Trisomy 2L
St 1-5
St 6-11
St 12-13
St 14-17
A1
A2
A3
A4
A5
A6
A7
A8
A9
A10
A11
A12
B
St 1-5
St 6-11
St 12-13
St 14-17
C
C1 Expression level of roX1 in embryo
C2 Expression level of roX1 in embryo
C3 Expression level of roX1 in embryo
C4 Expression level of roX1 in embryo
Relative fluorescence Intensity (RNA/ DAPI)
Wild Type
Trisomy 2L
MSL2-Trisomy 2L

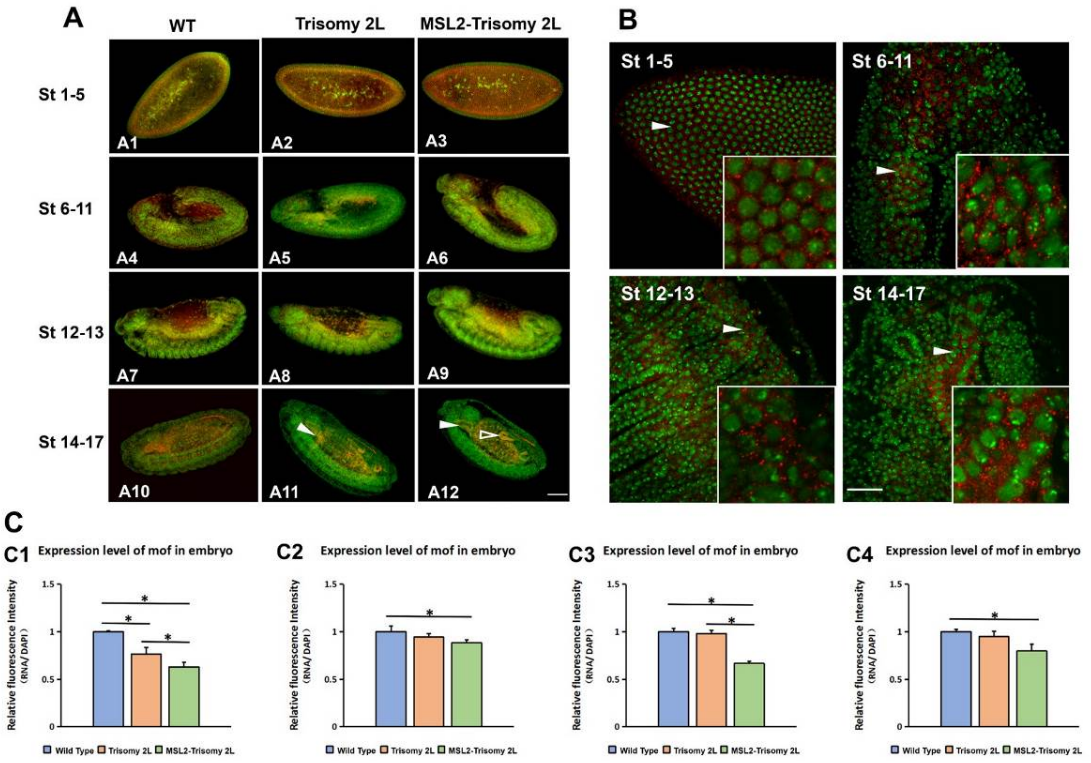
A
WT
Trisomy 2L
MSL2-Trisomy 2L
St 1-5
St 6-11
St 12-13
St 14-17
A1
A2
A3
A4
A5
A6
A7
A8
A9
A10
A11
A12
B
St 1-5
St 6-11
St 12-13
St 14-17
C
C1 Expression level of mof in embryo
C2 Expression level of mof in embryo
C3 Expression level of mof in embryo
C4 Expression level of mof in embryo
Relative fluorescence Intensity (RNA/ DAPI)
Wild Type
Trisomy 2L
MSL2-Trisomy 2L

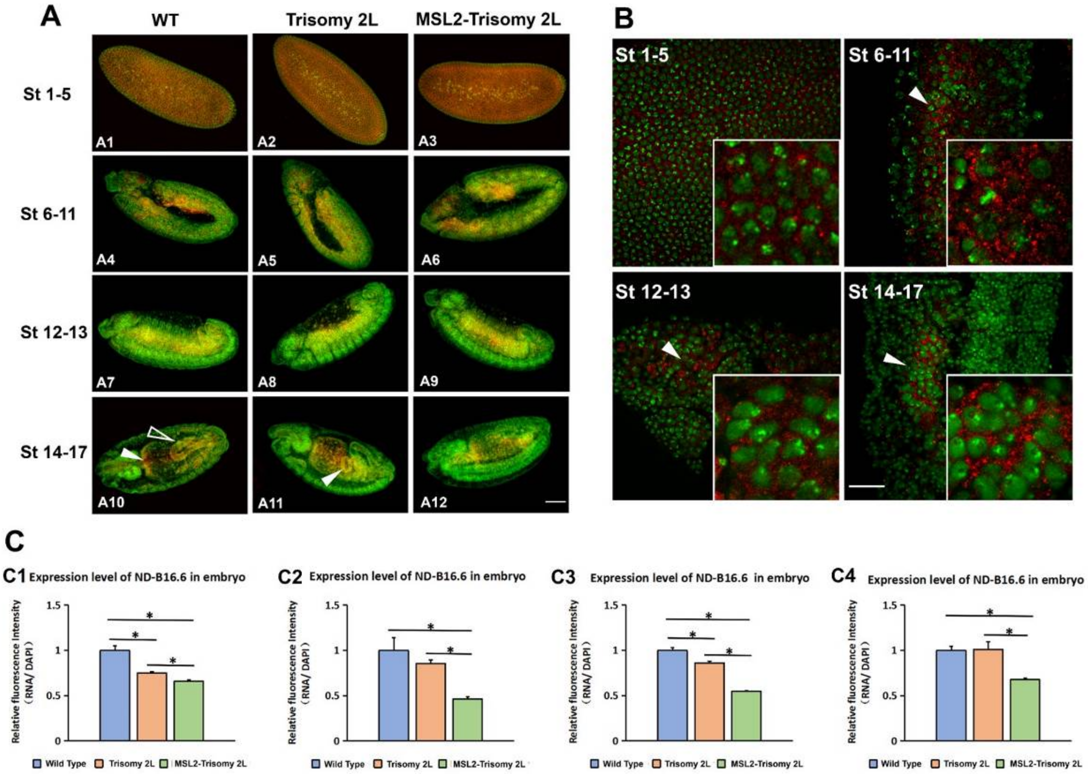
A
WT
Trisomy 2L
MSL2-Trisomy 2L
St 1-5
St 6-11
St 12-13
St 14-17
A1
A2
A3
A4
A5
A6
A7
A8
A9
A10
A11
A12
B
St 1-5
St 6-11
St 12-13
St 14-17
C
C1 Expression level of ND-B16.6 in embryo
C2 Expression level of ND-B16.6 in embryo
C3 Expression level of ND-B16.6 in embryo
C4 Expression level of ND-B16.6 in embryo
Relative fluorescence Intensity (RNA/ DAPI)
Wild Type
Trisomy 2L
MSL2-Trisomy 2L

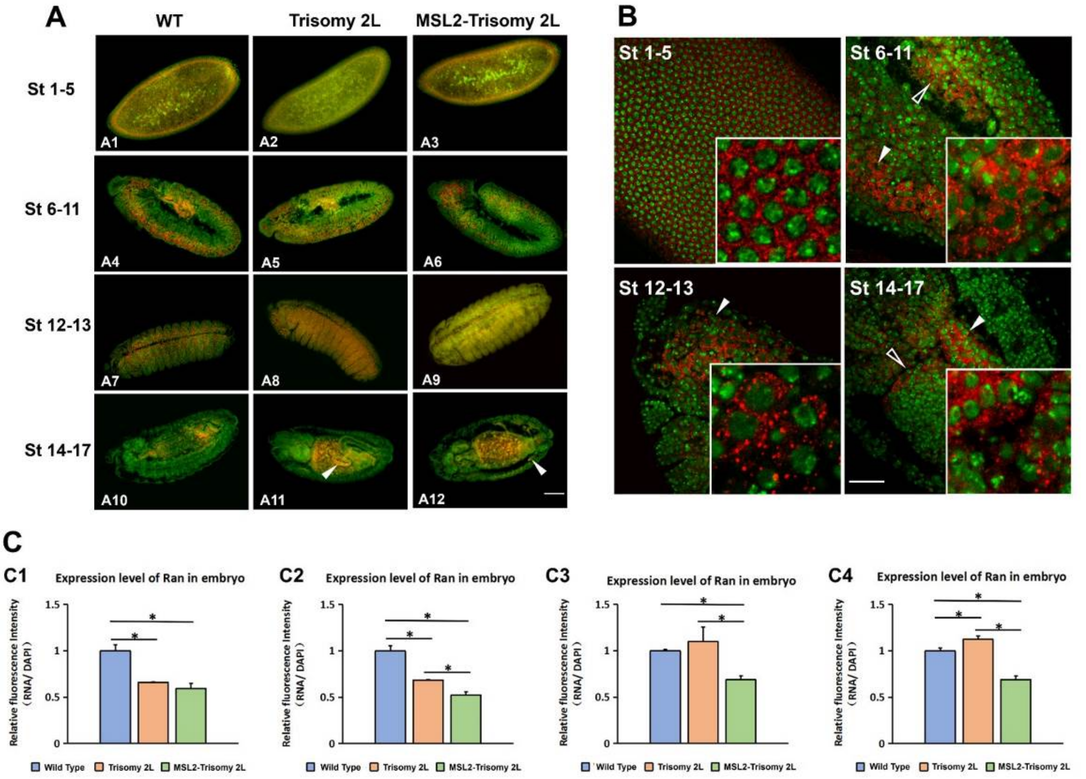
A
WT
Trisomy 2L
MSL2-Trisomy 2L
St 1-5
St 6-11
St 12-13
St 14-17
A1
A2
A3
A4
A5
A6
A7
A8
A9
A10
A11
A12
B
St 1-5
St 6-11
St 12-13
St 14-17
C
C1 Expression level of Ran in embryo
C2 Expression level of Ran in embryo
C3 Expression level of Ran in embryo
C4 Expression level of Ran in embryo
Relative fluorescence Intensity (RNA/ DAPI)
Wild Type
Trisomy 2L
MSL2-Trisomy 2L

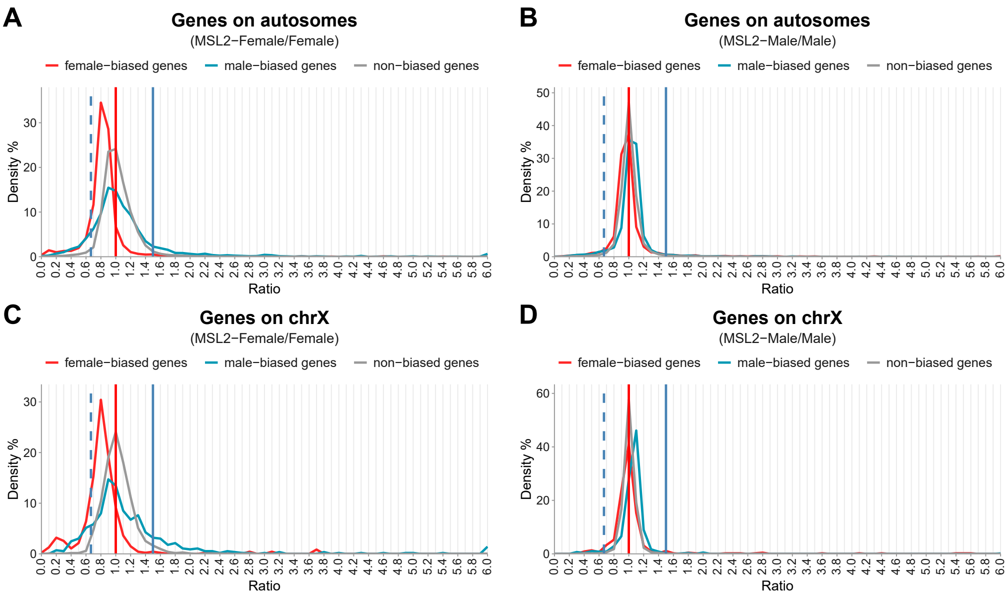

Figure S5. Ratio distributions in ectopically expressed MSL2 diploid. (**A**-**D**) Ratio distributions of sex-biased and non-sex-biased transcript isoforms in ectopically expressed MSL2 diploid compared with normal diploid in females (**A** and **C**) and males (**B** and **D**). All genes are divided into X (**A** and **B**) and autosomes (**C** and **D**) according to their positions on chromosomes. The vertical red solid line represents the ratio 1.00 (no change), the vertical blue solid line represents the ratio 1.50, and the vertical blue dashed line shows the ratio 0.67. The data set was obtained from GSE41570. The percentages of frequencies were plotted in bins of 0.1.

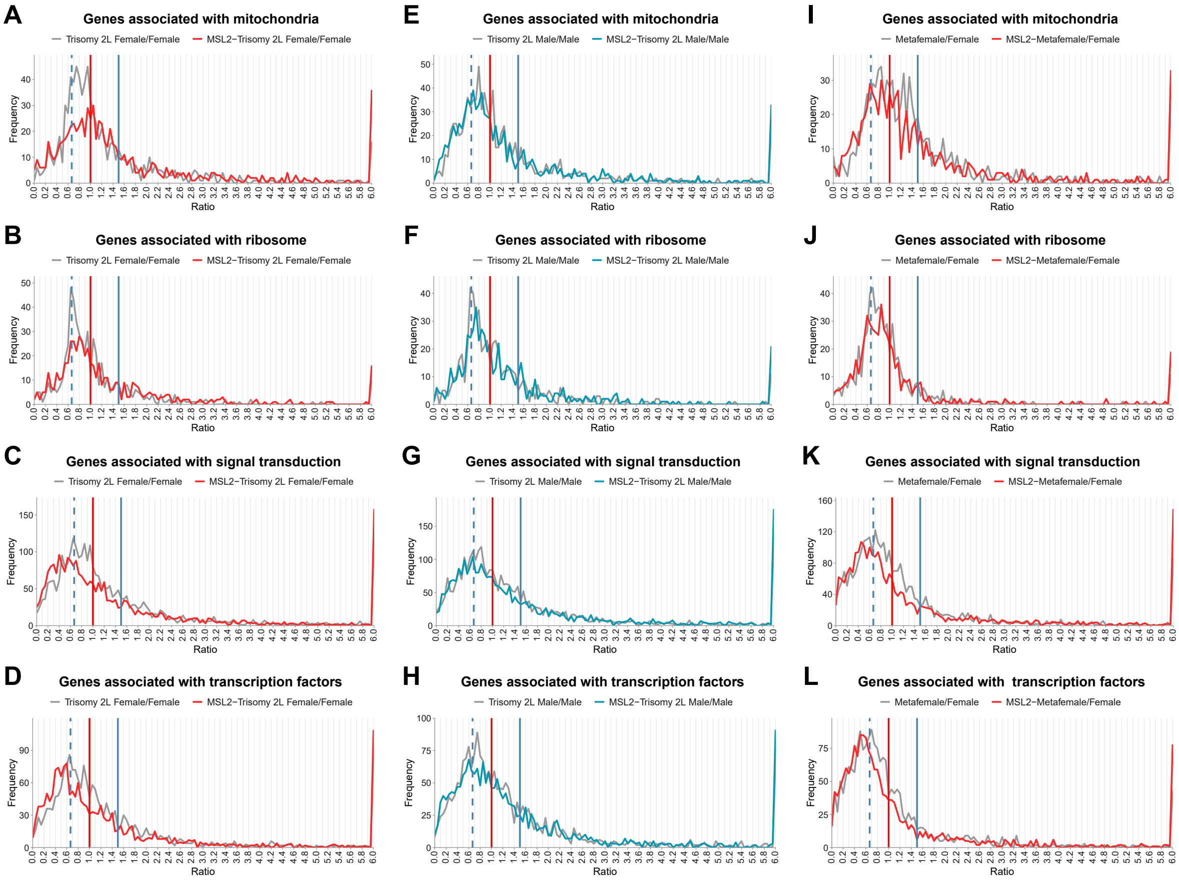

Figure S6. Ratio distributions of several functional categories of genes. (**A**-**L**) Ratio distributions of gene expression in trisomy and MSL2-trisomy compared with normal diploid in females (**A**-**D**), males (**E**-**H**) and metafemales (**I**-**L**). Genes associated with mitochondria (**A**, **E**, **I**), ribosome (**B**, **F**, **J**), signal transduction (**C**, **G**, **K**) and transcription factors (**D**, **H**, **L**) on all chromosomes are shown in the plots. The vertical red solid line represents the ratio 1.00 (no change), the vertical blue solid line represents the ratio 1.50 [the ratio of gene dosage effects (3/2)], and the vertical blue dashed line shows the ratio 0.67 [the ratio of inverse dosage effects (2/3)]. The gene list of mitochondria, ribosome and signal transduction were obtained based on gene GO annotation, and the transcription factors were obtained from AnimalTFDB 3.0. The ratio distributions were generated as described in *Methods* and the frequencies were plotted in bins of 0.05.

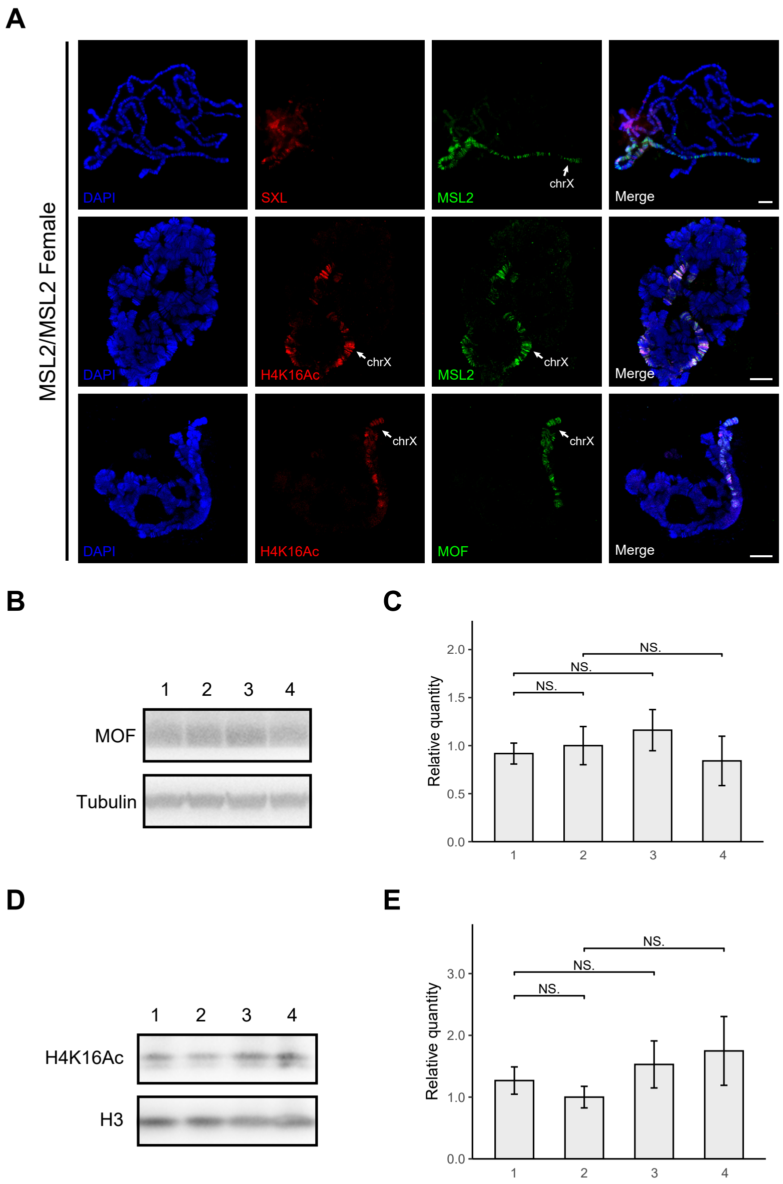

Figure S7. Characteristics of female *Drosophila* with ectopic expression of MSL2. (**A**) Immunofluorescence of *Drosophila* polytene chromosomes from third instar larvae of MSL2-females. The antibody of red and green channel is shown in the corresponding pictures. DNA is stained with DAPI in blue. Scale bars, 10 µm. (**B**) Western blot analysis of MOF in *Drosophila* with and without ectopic expression of MSL2. (**C**) Relative quantification of MOF in wild type and ectopically expressed MSL2 *Drosophila* based on western blot (N = 3). (**D**) Western blot analysis of H4K16Ac in *Drosophila* with and without ectopic expression of MSL2. (**E**) Relative quantification of H4K16Ac in wild type and ectopically expressed MSL2 *Drosophila* based on western blot (N = 3). Samples: 1, wild type female; 2, wild type male; 3, MSL2-female; 4, MSL2-male. Asterisk denotes a Student's *t* test P-value <0.05. The original images of western blot were provided in Figure S8.

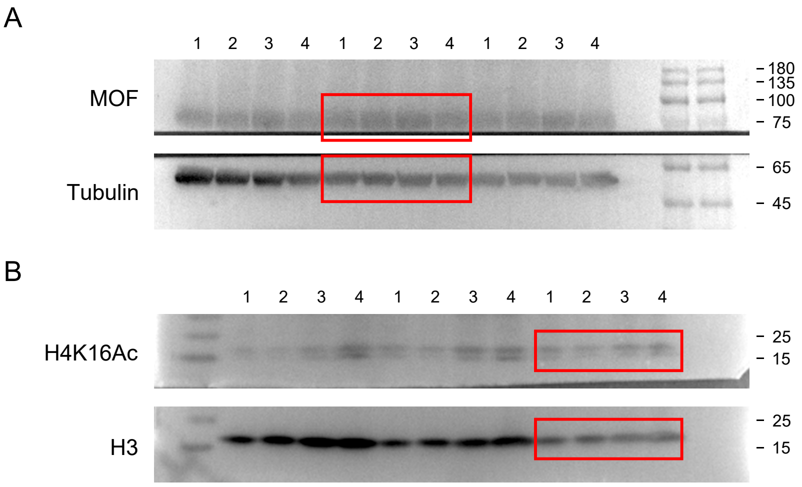

Figure S8. Western blot original images. (**A**) Western blot of MOF and Tubulin. (**B**) Western blot of H4K16Ac and H3. Samples: 1, wild type female; 2, wild type male; 3, MSL2-female; 4, MSL2-male. Antibodies: anti-MOF (Santa Cruz, sc-22351); anti-Tubulin (Proteintech, 66031); anti-H4K16Ac (EMD Millipore, 07-329); anti-Histone H3 (Easybio, BE3015). Western blot was performed as described in the previously published article [1]. Images were captured using Tanon 5200Multi Chemiluminescence Image Analysis System. The regions used in Figure S7 were denoted by red boxes.

Additional References

[1] Brown, E. J. & Bachtrog, D. The chromatin landscape of *Drosophila*: comparisons between species, sexes, and chromosomes. *Genome Res*. **24**, 1125-1137, doi:10.1101/gr.172155.114 (2014).
